# HSA-Binding Prodrugs-Based Nanoparticles Endowed with Chemo and Photo-Toxicity against Breast Cancer

**DOI:** 10.3390/cancers14040877

**Published:** 2022-02-10

**Authors:** Valentina Rapozzi, Francesca Moret, Luca Menilli, Andrea Guerrini, Daniele Tedesco, Marina Naldi, Manuela Bartolini, Mariachiara Gani, Sonia Zorzet, Marta Columbaro, Celeste Milani, Cecilia Martini, Claudia Ferroni, Greta Varchi

**Affiliations:** 1 Department of Medicine, University of Udine, 33100 Udine, Italy; valentina.rapozzi@uniud.it (V.R.); gani.mariachiara@spes.uniud.it (M.G.); 2 Department of Biology, University of Padova, 35100 Padova, Italy; francesca.moret@unipd.it (F.M.); luca.menilli@unipd.it (L.M.); celeste.milani@studenti.unipd.it (C.M.); 3 Institute of Organic Synthesis and Photoreactivity, ISOF-CNR, 40129 Bologna, Italy; andrea.guerrini@isof.cnr.it (A.G.); daniele.tedesco@isof.cnr.it (D.T.); cecilia.martini@isof.cnr.it (C.M.); 4 Department of Pharmacy and Biotechnology Alma Mater Studiorum, University of Bologna, 40126 Bologna, Italy; marina.naldi@unibo.it (M.N.); manuela.bartolini3@unibo.it (M.B.); 5 Department of Life Sciences, University of Trieste, 34127 Trieste, Italy; zorzet@units.it; 6 Electron Microscopy Platform, IRCCS Istituto Ortopedico Rizzoli, 40136 Bologna, Italy; marta.columbaro@ior.it

**Keywords:** combination therapy, breast cancer, prodrugs, pheophorbide a, paclitaxel, albumin-binding, nanoparticles

## Abstract

**Simple Summary:**

In this study, we developed novel bioresponsive HSA-binding nanoparticles co-delivering paclitaxel (PTX) prodrugs and the photosensitizer pheophorbide a (Pba) for the combined photo- and chemo-treatment of breast cancer. Extensive structural characterization allowed us to evaluate the size, stability and morphology of nanoparticles, which exhibited sustained and controlled drug release under the distinctive redox conditions of the tumor environment. HSA-binding PTX/Pba nanoparticles showed higher Pba uptake in human breast cancer cells and a synergistic antitumor effect upon light irradiation. Preliminary in vivo experiments using low drug doses showed the potential of our bioresponsive nanoparticles to reduce the primary tumor mass while diminishing the number of lung metastases, thus suggesting the effectiveness of this novel approach.

**Abstract:**

Exploiting the tumor environment features (EPR effect, elevated glutathione, reactive oxygen species levels) might allow attaining a selective and responsive carrier capable of improving the therapeutic outcome. To this purpose, the in situ covalent binding of drugs and nanoparticles to circulating human serum albumin (HSA) might represent a pioneering approach to achieve an effective strategy. This study describes the synthesis, in vitro and in vivo evaluation of bioresponsive HSA-binding nanoparticles (**MAL**-PTX_2_S@Pba), co-delivering two different paclitaxel (PTX) prodrugs and the photosensitizer pheophorbide a (Pba), for the combined photo- and chemo-treatment of breast cancer. Stable and reproducible **MAL**-PTX_2_S@Pba nanoparticles with an average diameter of 82 nm and a PTX/Pba molar ratio of 2.5 were obtained by nanoprecipitation. The in vitro 2D combination experiments revealed that **MAL**-PTX_2_S@Pba treatment induces a strong inhibition of cell viability of MDA-MB-231, MCF7 and 4T1 cell lines, whereas 3D experiments displayed different trends: while **MAL**-PTX_2_S@Pba effectiveness was confirmed against MDA-MB-231 spheroids, the 4T1 model exhibited marked resistance. Lastly, despite using a low PTX-PDT regimen (e.g., 8.16 mg/Kg PTX and 2.34 mg/Kg Pba), our formulation showed to foster primary tumor reduction and curb lung metastases growth in 4T1 tumor-bearing mice, thus setting the basis for further preclinical validations.

## 1. Introduction

Extensive angiogenesis, hyper-vascularization, defective vascular architecture, and impaired lymphatic drainage are common features of the pathophysiology of solid tumors, resulting in the so-called Enhanced Permeability and Retention effect (EPR). These characteristics are central pillars for the development of tumor-selective drug delivery systems using macromolecules and nanoparticles as carriers [1].

Owing to the impressive progress in materials science and nanotechnology, a large variety of nanocarriers has been generated in the last decades to enhance the delivery of toxic and highly hydrophobic drugs, as well as to co-deliver multiple synergic drugs at the site of action [2,3]. Despite the promising outcomes in pre-clinical investigations, their translation to the clinic is still challenging, primarily due to difficulties arising during the up-scaling process, reflecting in regulatory ambiguities [4], and for the use of highly specific and expensive technologies that often leads to a limited benefit to patients [5,6,7].

Human serum albumin (HSA) is the most abundant blood plasma protein, with an average half-life of three weeks and a hydrodynamic radius of around 7 nm, that has shown great potential for drug delivery thanks to its physiological role as a carrier protein for endogenous and exogenous substances [8,9]. HSA has been employed to form nanoparticles (NPs) with different anticancer drugs, enhancing their delivery at the tumor tissues thanks to the EPR effect and to the over-expression of specific HSA-binding proteins, such as gp60 and SPARC (secreted protein acidic and rich in cysteine) [10]. These proteins play an essential role in the selective internalization and accumulation of HSA-based NPs and HSA-bound drugs into the tumor interstitium [9,11], making HSA an attractive, safe and inexpensive molecule for the preparation of nano-structured carriers for drugs to enhance their circulation half-life and targeting.

Two major approaches have been applied to exploit HSA in drug delivery systems. In the former, HSA–drug NPs are prepared through different techniques (i.e., desolvation, emulsification, self-assembly, thermal-gelation, etc.) [12], and then injected in the body intravenously. Among HSA-based NPs, the most successful example is represented by Abraxane^®^, a formulation of the anticancer drug paclitaxel (PTX) obtained through the NAB™ technology, a high-pressure emulsion procedure [13]. As for the second approach, HSA-binding peptides, drugs or prodrugs are exogenously prepared and administered intravenously for in situ binding to circulating HSA [8,14]. In the latter case, drugs or prodrugs are specifically designed to preferentially bind to HSA, which possesses at least 7 binding sites for fatty acids, 3 binding sites for drugs and a single cysteine residue (Cys34) not involved in the network of 17 intramolecular disulfide bonds. These peculiar features of HSA can be exploited as convenient targets of non-covalent or covalent interactions with the protein [15]. Approximately 70% of the circulating HSA bears the freely accessible Cys34 residue in reduced form (mercaptoalbumin), accounting for 80–90% of the total thiol concentration in human plasma (400–500 μM) [16,17]. The in situ binding of drugs or prodrugs to circulating HSA might offer several advantages over the use of pre-formed HSA-based NPs in terms of system reproducibility, scalability, and regulatory requirements. To this purpose, the maleimide group has been proposed for the fast and effective covalent binding to Cys34 on HSA [18,19].

Cancer is known to develop via a multistep carcinogenesis process entailing numerous cellular and physiological events that make it a highly complex disease. The most common cancer treatments are limited to chemotherapy, radiation and surgery; despite many advances in these conventional treatment options, cancer therapy is still far from optimal because of overwhelming drawbacks [20]. In this scenario, the use of unconventional treatment modalities has gained increasing attention in the last decades [21]. Among these, light-triggered therapies such as photodynamic therapy (PDT) are very fascinating, since light is a powerful means for the local and non-invasive production of therapeutic agents at a desired site [22,23] through the controlled and timely dosage of the released species. PDT has already been approved for the treatment of superficial cancers and is currently under preclinical and clinical investigation for other solid tumors, such as osteosarcoma, cervix epithelioid carcinoma, prostate and breast cancers [24,25]. In PDT, cancer cells are destroyed by reactive oxygen species (ROS), formed upon the illumination of a photosensitizer (PS) at a specific wavelength in the presence of oxygen and/or biomolecules [26,27,28]. To enable a better therapeutic outcome, novel concepts of combining PDT and chemotherapy have been developed by means of nano-technological approaches [29,30,31], which have been shown to overcome the limitations encountered by each therapy when used alone. For example, the combination of PDT with chemotherapy has the potential to induce antitumor immunity [32,33] or revert multi-drug resistance induced by the chemotherapeutic agent [34,35].

In the present paper, we describe for the first time the synthesis, as well as the in vitro and in vivo evaluation, of bioresponsive HSA-binding NPs co-delivering PTX prodrugs and the PS pheophorbide a (Pba) for the combined photo- and chemo-treatment of breast cancer. Preparation of the nanoparticles was achieved by nanoprecipitation of a bioresponsive HSA-binding conjugate of PTX, Pba and different amounts of a PTX dimeric prodrug [30], allowing the fine tuning of the relative amount of the two active principles.

Importantly, both the thioether (PTX dimer) and the disulfide bond (HSA-binding PTX conjugate) are designed to selectively release PTX under the reducing conditions typical of the tumor environment, characterized by elevated glutathione (GSH) concentrations and high levels of ROS [36]. Of note, our approach should allow three levels of treatment selectivity owing to: (i) the use of HSA as endogenous carrier of nanoparticles; (ii) the use of PTX prodrugs capable of preferentially releasing the drug within the tumor; (iii) the intrinsic selective action of PDT.

To prove the rationale of our approach, HSA-binding and non-binding nanoparticles were prepared, and their behavior was compared both in vitro and in vivo. HSA-binding nanoparticles are endowed with a maleimide group, while the corresponding non-has-binding nanoparticles are decorated with a tert-butoxyl moiety, which is incapable of covalent interaction with Cys34.

## 2. Materials and Methods

### 2.1. Materials

All reagents were used as obtained from commercial sources unless otherwise indicated. Solvents were dried over standard drying agents and freshly distilled prior to use.

#### Paclitaxel (PTX) Was Purchased from TCI Europe

Pheophorbide a (Pba) was purchased from Cayman Chemical (Ann Arbor, MI, USA) and used without further purification. Linear heterobifunctional polyethylene glycol (PEG) derivatives, namely tert-butoxycarbonyl amine PEG-45 amine hydrochloride (**BOC**-NH-PEG_45_-NH_2_·HCl) and maleimide PEG-45 amine trifluoroacetic acid salt (**MAL**-PEG_45_-NH_2_·TFA) were purchased from JenKem Technology USA (Plano, TX, USA). Deuterated solvents chloroform-*d* (CDCl_3_) and dimethyl sulfoxide-*d*_6_ (DMSO-*d*_6_) were purchased from Euriso-Top (Saint-Aubin, France). High-performance liquid chromatography (HPLC)-grade acetonitrile (ACN) and trifluoroacetic acid (TFA) were purchased from Fisher Scientific (Milan, Italy). Cyclohexane (Cy), ethyl acetate (EA), dichloromethane (DCM), triethylamine (TEA), acetone, dimethyl sulfoxide (DMSO), tetrahydrofuran (THF), human serum albumin (HSA; fraction V, product code A1653), bovine serum albumin (BSA) and all other chemicals used for the synthesis and characterization of compounds and NPs were purchased from Sigma-Aldrich (Milan, Italy), unless otherwise specified. Foetal bovine serum (FBS) catalogue number 10270, lot number 2337968 was purchased from Life Technology (Monza, Italy). Ultrapure water was produced using a Sartorius Arium Pro^®^ system (Sartorius, Monza, Italy).

Proton nuclear magnetic resonance (^1^H NMR) spectra were recorded on 400 and 500 MHz Varian spectrometers. ^1^H chemical shifts values (δ) are referenced to the residual non-deuterated components of NMR solvents. Flash chromatography was performed on Teledyne Isco CombiFlash Rf 200 using RediSep normal-phase silica flash columns (230–400 mesh). Thin-layer chromatography (TLC) was performed on silica gel 60 F254 plastic sheets. The purity of all compounds was >90%, as determined by HPLC–UV analysis (for experimental details, see Section 2.5), unless otherwise stated. Absorption spectra were recorded using a Cary 100 UV–Vis spectrophotometer (Agilent Technologies, Milan, Italy).

### 2.2. Cell Lines

MDA-MB-231 (human triple negative breast cancer), MCF7 (human breast adenocarcinoma), 4T1 (mouse triple negative breast cancer) and MCF10A (human non-malignant breast epithelial) cell lines were purchased from American Type Culture Collection (ATCC, Rockville, MD, USA). MDA-MB-231 and MCF7 cells were grown in Dulbecco’s Modified Eagle Medium (DMEM) with Glutamax^TM^ supplemented with 10% heat inactivated FBS, 100 U/mL streptomycin, 100 µg/mL penicillin G as antibiotics. 4T1 cells were grown in RPMI 1640 ATCC formulated medium supplemented with 10% FBS and antibiotics, whereas MCF10A cells were cultured in DMEM/F12 medium supplemented with 5% horse serum, 20 ng/mL EGF, 0.5 mg/mL hydrocortisone, 100 ng/mL cholera toxin, 10 μg/mL insulin and antibiotics. Cell culture medium and supplements were purchased from Life Technologies or Sigma-Aldrich (Italy), while sterile plasticware was purchased from Falcon^®^ (Corning, New York, NY, USA).

### 2.3. Synthesis and Characterization of PTX Prodrugs

#### 2.3.1. Synthesis of Compound **2**

**Scheme 1:**
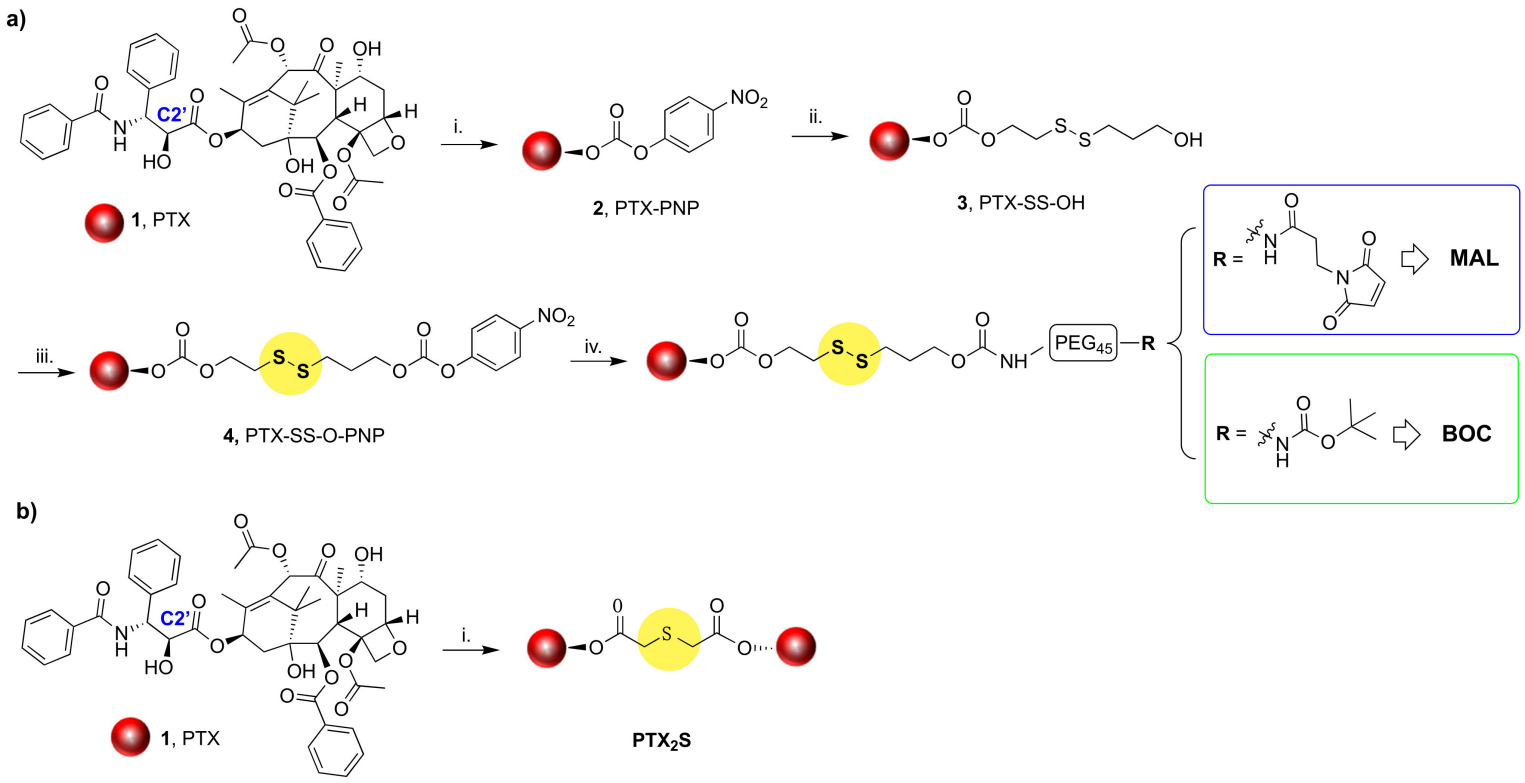
(**a**) Synthesis of HSA-binding and non-binding PTX prodrugs: i. 4-Nitrophenyl chloroformate, DMAP, TEA, DCM, RT, Ar, on, Y = 56%; ii. 2-Hydroxyethyl disufide, DMAP, THF/DCM, RT; Ar, 5 h, Y = 90%; iii. 4-Nitrophenyl chloroformate, DMAP, TEA, DCM, RT, Ar, on, Y = 55%; iv. A--NH3+-PEG45-R, TEA, DCM, RT, Ar, on Y = 69–73%. (**b**) Synthesis of PTX dimeric prodrug: i. 2,2’-thiodiacetic acid, EDC, DMAP, DCM, RT, Ar, 4 h 30’, Y = 88%.

PTX (**1**, Scheme 1; 0.36 mmol, 1 eq.) was dissolved in 3.5 mL of dry DCM, followed by the addition of 4-(dimethylamino)pyridine (DMAP; 0.036 mmol, 0.1 eq.) and TEA (0.36 mmol, 1 eq.). A solution of 4-nitrophenyl chloroformate (PNP-Cl; 0.47 mmol, 1.3 eq.) dissolved in 2.5 mL of dry DCM was added dropwise to the reaction mixture, that was stirred under argon atmosphere at room temperature overnight. The crude product was purified by flash chromatography (eluent: Cy/EA, 70:30 → 50:50, *v*/*v*) to obtain intermediate **2** as a white solid in 56% yield.

^1^H NMR (500 MHz, CDCl_3_) δ 8.26 (d, *J* = 9.1 Hz, 2H), 8.16 (d, *J* = 7.5 Hz, 2H), 7.76 (d, *J* = 7.6 Hz, 2H), 7.61 (t, *J* = 7.4 Hz, 1H), 7.56–7.39 (m, 11H), 7.35 (d, *J* = 9.1 Hz, 2H), 6.94 (d, *J* = 9.4 Hz, 1H), 6.34 (t, *J* = 9.1 Hz, 1H), 6.30 (s, 1H), 6.11 (dd, *J* = 9.3, 1.8 Hz, 1H), 5.71 (d, *J* = 7.1 Hz, 1H), 5.55 (d, *J* = 2.4 Hz, 1H), 5.01–4.94 (m, 1H), 4.43 (dt, *J* = 15.5, 7.7 Hz, 1H), 4.33 (d, *J* = 8.5 Hz, 1H), 4.22 (d, *J* = 8.4 Hz, 1H), 3.83 (d, *J* = 7.2 Hz, 1H), 2.61–2.40 (m, 6H), 2.31–2.18 (m, 4H), 1.93 (s, 3H), 1.69 (s, 3H), 1.26 (s, 3H), 1.16 (s, 3H). HRMS (ESI, *m*/*z*): calculated for C_54_H_54_N_2_O_18_ [M + H]^+^ 1019.3450, found 1019.3453.

#### 2.3.2. Synthesis of Compound **3**

2-Hydroxyethyl disulfide (0.18 mmol, 1.9 eq.), dissolved in 1.5 mL of dry THF, was added dropwise at room temperature to a solution of **2** (0.1 mmol, 1 eq.) and DMAP (0.01 mmol, 0.1 eq.) in 2 mL of dry DCM, under argon atmosphere. The mixture was stirred for 5 h and the progress of the reaction was monitored by TLC. The crude oil was then purified by flash chromatography (eluent: DCM/acetone, 90:10, *v*/*v*) to obtain intermediate **3** as a white solid in 90% yield.

^1^H NMR (500 MHz, CDCl_3_) δ 8.14 (d, *J* = 7.3 Hz, 2H), 7.75 (d, *J* = 7.3 Hz, 2H), 7.61 (t, *J* = 7.4 Hz, 1H), 7.56–7.31 (m, 11H), 7.09 (d, *J* = 9.4 Hz, 1H), 6.34–6.20 (m, 2H), 6.01 (dd, *J* = 9.3, 2.6 Hz, 1H), 5.69 (d, *J* = 7.1 Hz, 1H), 5.44 (d, *J* = 2.7 Hz, 1H), 4.97 (d, *J* = 8.3 Hz, 1H), 4.49–4.34 (m, 3H), 4.32 (d, *J* = 8.5 Hz, 1H), 4.21 (d, *J* = 8.5 Hz, 1H), 3.82 (d, *J* = 6.4 Hz, 2H), 2.95 (t, *J* = 6.5 Hz, 2H), 2.64–2.33 (m, 6H), 2.29–2.20 (m, 5H), 1.92 (s, 3H), 1.75 (s, 1H), 1.68 (s, 3H), 1.24 (s, 3H), 1.13 (s, 3H). MS (ESI, *m*/*z*): 1034.2 [M + H]^+^, 1056.2 [M + Na]^+^.

#### 2.3.3. Synthesis of Compound 4

Compound 3 (0.071 mmol, 1 eq.) was dissolved in 1.5 mL of anhydrous DCM, followed by the addition of DMAP (0.007 mmol, 0.1 eq.) and TEA (0.071 mmol, 1 eq.). Then, a solution of PNP-Cl (0.085 mmol, 1.2 eq.) in 0.5 mL of anhydrous DCM was added dropwise to the reaction mixture, that was stirred under argon atmosphere at room temperature overnight. The crude material was purified by flash chromatography (eluent: DCM/EA, 90:10 → 85:15, *v*/*v*) to obtain intermediate **4** in 55% yield.

^1^H NMR (400 MHz, CDCl_3_) δ 8.23 (d, *J* = 1.8 Hz, 2H), 8.14 (d, *J* = 7.3 Hz, 2H), 7.71 (d, *J* = 7.3 Hz, 2H), 7.61 (t, *J* = 7.4 Hz, 1H), 7.55–7.31 (m, 12H), 6.98 (d, *J* = 9.4 Hz, 1H), 6.36–6.21 (m, 2H), 6.01 (dd, *J* = 9.4, 2.6 Hz, 1H), 5.69 (d, *J* = 7.1 Hz, 1H), 5.45 (d, *J* = 2.5 Hz, 1H), 4.97 (d, *J* = 8.0 Hz, 1H), 4.57–4.34 (m, 4H), 4.32 (d, *J* = 8.4 Hz, 1H), 4.20 (d, *J* = 8.5 Hz, 1H), 3.82 (d, *J* = 7.0 Hz, 1H), 3.04–2.85 (m, 2H), 2.63–2.36 (m, 6H), 2.30–2.19 (m, 5H), 1.92 (m, 3H), 1.75 (s, 1H), 1.66 (s, 3H), 1.24 (s, 3H), 1.14 (s, 3H).

#### 2.3.4. Synthesis of PTX-SS-PEG-**MAL** (**5**, **MAL**)

TEA (0.0309, 3.6 eq.) was added to a solution of **MAL**-PEG_45_-NH_2_·TFA (0.0103 mmol, 1.2 eq.) dissolved in 1 mL of dry DCM under argon atmosphere. After 30 min, compound **4** (0.0086 mmol, 1 eq.) was added dropwise to the mixture and stirred overnight at room temperature. The reaction mixture was concentrated, and the solid was then dissolved in 1 mL of DMSO and purified through dialysis (Tube-O-DIALYZER mini dialysis system, MCWO: 1 kDa) against ultrapure water for 24 h; the external medium was replaced with fresh ultrapure water every 4 h. The purified solution containing the PTX conjugate PTX-SS-PEG-**MAL** (**5**, **MAL**) was freeze-dried into powder affording the desired product in 73% yield.

^1^H NMR (500 MHz, DMSO-*d*_6_) δ 9.28 (d, *J* = 8.5 Hz, 1H), 8.34–8.28 (m, 1H), 8.09 (d, *J* = 3.3 Hz, 1H), 7.97 (d, *J* = 7.5 Hz, 2H), 7.84 (d, *J* = 7.3 Hz, 2H), 7.77–7.59 (m, 4H), 7.59–7.52 (m, 2H), 7.52–7.40 (m, 6H), 7.21–7.14 (m, 1H), 6.99 (s, 2H), 6.95–6.89 (m, 1H), 6.28 (s, 1H), 5.82 (t, *J* = 9.0 Hz, 1H), 5.74 (s, 1H), 5.54 (t, *J* = 8.6 Hz, 1H), 5.41 (d, *J* = 7.1 Hz, 1H), 5.36 (d, *J* = 8.8 Hz, 1H), 4.89 (d, *J* = 7.2 Hz, 2H), 4.63 (s, 1H), 4.47–4.33 (m, 2H), 4.17–4.05 (m, 2H), 4.00 (q, *J* = 8.3 Hz, 2H), 3.67–3.61 (m, 2H), 3.49 (s, 180H), 3.40–3.33 (m, 4H), 3.18–3.02 (m, 6H), 2.99 (t, *J* = 6.2 Hz, 2H), 2.89 (t, *J* = 6.1 Hz, 1H), 2.25 (s, 3H), 2.09 (d, *J* = 4.3 Hz, 3H), 1.78 (s, 3H), 1.49 (s, 3H), 1.04–0.96 (m, 3H).

#### 2.3.5. Synthesis of PTX-SS-PEG-**BOC** (**6**, **BOC**)

The PTX conjugate PTX-SS-PEG-**BOC** (**6**, **BOC**) was prepared by replacing **MAL**-PEG_45_-NH_2_·TFA with **BOC**-NH-PEG_45_-NH_2_·HCl and following the conditions previously reported for compound **5** (**MAL**) with 69% yield.

^1^H NMR (500 MHz, DMSO-*d*_6_) δ 9.28 (d, *J* = 8.5 Hz, 1H), 8.34–8.28 (m, 1H), 8.09 (d, *J* = 3.3 Hz, 1H), 7.97 (d, *J* = 7.5 Hz, 2H), 7.84 (d, *J* = 7.3 Hz, 2H), 7.77–7.59 (m, 4H), 7.59–7.52 (m, 2H), 7.52–7.40 (m, 6H), 7.21–7.14 (m, 1H), 6.95–6.89 (m, 1H), 6.28 (s, 1H), 5.82 (t, *J* = 9.0 Hz, 1H), 5.74 (s, 1H), 5.54 (t, *J* = 8.6 Hz, 1H), 5.41 (d, *J* = 7.1 Hz, 1H), 5.36 (d, *J* = 8.8 Hz, 1H), 4.89 (d, *J* = 7.2 Hz, 2H), 4.63 (s, 1H), 4.47–4.33 (m, 2H), 4.17–4.05 (m, 2H), 4.00 (q, *J* = 8.3 Hz, 2H), 3.67–3.61 (m, 2H), 3.49 (s, 180H), 3.40–3.33 (m, 4H), 3.18–3.02 (m, 6H), 2.99 (t, *J* = 6.2 Hz, 2H), 2.89 (t, *J* = 6.1 Hz, 1H), 2.25 (s, 3H), 2.09 (d, *J* = 4.3 Hz, 3H), 1.78 (s, 3H), 1.49 (s, 3H), 1.36 (s, 9H), 1.04–0.96 (m, 3H).

#### 2.3.6. PTX Dimer (PTX_2_S)

The PTX dimer (PTX_2_S) was synthesized as previously reported [37]. Briefly, PTX (0.12 mmol, 1 eq.) was dissolved in DCM (2.0 mL) then 2,2′-thiodiacetic acid (0.085 mmol, 0.73 eq), 1-ethyl-3-(3-dimethylaminopropyl)carbodiimide hydrochloride (EDC·HCl; 0.24 mmol, 2 eq.) and DMAP (0.012 mmol, 0.1 eq) were added under argon atmosphere. After stirring for 1h at room temperature, an additional 23 mg of EDC·HCl (0.12 mmol, 1 eq) and 1.5 mg of DMAP (0.012 mmol, 0.1 eq) were added. The resulting mixture was stirred at room temperature for further 4h and the crude material was purified by flash chromatography (eluent: DCM/EA, 70:30, *v*/*v*) to afford PTX_2_S as a white solid in 88%.

^1^H NMR (500 MHz, CDCl_3_) δ 8.16 (d, *J* = 7.4 Hz, 4H), 7.74 (d, *J* = 7.6 Hz, 4H), 7.66–7.58 (m, 2H), 7.56–7.49 (m, 4 H), 7.48–7.31 (m, 16 H), 7.26–7.21 (m, NH, 2 H), 6.34–6.19 (m, 4 H), 6.09 (dd, *J* = 9.3, 2.5 Hz, 2 H), 5.68 (d, *J* = 7.1 Hz, 2 H), 5.53 (d, *J* = 2.9 Hz, 2 H), 4.98 (d, *J* = 8.6 Hz, 2 H), 4.43 (dd, *J* = 10.8, 6.7 Hz, 2 H), 4.32 (d, *J* = 8.4 Hz, 2 H), 4.21 (d, *J* = 8.4 Hz, 2 H), 3.81 (d, *J* = 7.0 Hz, 2 H), 3.27 (d, *J* = 14.7. Hz, 2 H), 3.16 (d, *J* = 14.7. Hz, 2 H), 2.63–2.52 (m, 2 H), 2.49 (s, 6 H), 2.39 (dd, *J* = 15.3, 9.4 Hz, 2 H), 2.22 (s, 6 H), 2.19–2.12 (m, 2 H), 1.89 (s, 6 H), 1.69 (s, 6 H), 1.22 (s, 6 H), 1.14 (s, 6 H).

### 2.4. Preparation of NPs

All nanoparticles were obtained by the nanoprecipitation method. As representative example for the preparation of **MAL**-PTX_2_S@Pba nanoparticles, a DMSO solution of **MAL** (3.6 mg, 120 μL, 30 mg/mL), a DMSO solution of PTX_2_S (2.02 mg, 68 μL, 30 mg/mL) and a freshly prepared DMSO solution of Pba (0.78 mg, 520 μL, 1.5 mg/mL), were simultaneously and slowly injected into 4.8 mL of ultrapure water at ambient temperature under vigorous stirring to afford the desired nanoparticles at room temperature.

nPTX_2_S were also prepared by nanoprecipitation as previously reported [30]. Briefly, 50 μL of a PTX_2_S solution in DMSO (10 mg/mL) were slowly injected into 0.8 mL of ultrapure water, under vigorous stirring. After 15 min, all NPs’ solution were analyzed through dynamic light scattering (DLS, 200 μL of NPs’ solution in 1.6 mL of water) and then suspensions were stored at 0 °C. To remove excess DMSO and unloaded Pba, NP solutions were purified through different cycles (×4–5 times) of ultrafiltration (Amicon Ultra Centrifugal Filter, MWCO: 1000 kDa, 8000 rpm, 3 min), till the volume was reduced to the desired concentration and the organic solvent completely removed. As reported in our previous work, the ultrafiltration process does not affect the hydrodynamic diameters and polydispersity of nanoparticles, confirming their excellent stability [30].

### 2.5. Characterization of NPs

The hydrodynamic diameter and polydispersity index (PDI) of nanoparticles in aqueous solution (0.27 mg/mL) were determined by DLS analysis at 25 °C using a NanoBrook Omni Particle Size Analyzer (Brookhaven Instruments Corporation, New York, NY, USA) equipped with a 35 mW red diode laser (nominal wavelength 640 nm). Electrophoretic mobility, i.e., ζ-potential, was measured at 25 °C using the same instrument.

Morphology of nanoparticles was analyzed by transmission electron microscopy (TEM). Nanoparticles (0.1 mg/mL) were dispensed as a drop on a carbon-coated nickel grid and after 20 min, any excess of the solution was absorbed by filter paper. The nanoformulation was subsequently observed with a Jeol Jem-1011 transmission electron microscope (Jeol Jem, Peabody, MA, USA).

In vitro stability studies were performed over time (72 h) at 37 °C in (i) in phosphate buffered saline (PBS; pH 7.4), (ii) FBS 20% in PBS pH 7.4, *v*/*v*, and (iii) HSA 35 mg/mL in PBS pH 7.4. All experiments were carried out in the dark to avoid possible disassembly contribution due to ROS formation under natural light exposure. In a typical experiment, 0.2 mL of nanoparticles’ solution (1.3 mg/mL) were diluted with 0.8 mL of the selected stability medium, while maintaining them at 37 °C. Changes in particle’s size distribution during time were monitored by DLS.

### 2.6. Release Kinetics of PTX and Pba from **MAL**-PTX_2_S@Pba NPs

The release kinetics of PTX and Pba from **MAL**-PTX_2_S@Pba NPs was evaluated in different redox conditions by equilibrium dialysis. **MAL**-PTX_2_S@Pba NPs (5.51 mL in water/DMSO, 87:13, *v*/*v*) were prepared as described previously and contained 3.60 mg **MAL**, 2.02 mg PTX_2_S and 0.78 mg Pba. **MAL**-PTX_2_S@Pba NPs were then split into five samples (0.75 mL), loaded inside 21-mm cellulose membrane dialysis tubes (MWCO: 12 kDa; Sigma-Aldrich, Milan, Italy) and dialyzed at pH 7.4 against 10 mL of five different release media based on the same solvent mixture (PBS/EtOH, 70:30, *v*/*v*): (A) redox-neutral (no additives); (B) mildly reducing (10 μM DTT), mimicking physiological condition; (C) strongly reducing (10 mM DTT), mimicking tumor environment condition; (D) mildly oxidizing (2 mM H_2_O_2_), mimicking physiological condition; (E) strongly oxidizing (10 mM H_2_O_2_), mimicking tumor environment condition.

Dialysis experiments were carried out at controlled temperature (37 °C) over 49 h under gentle agitation using a M 200-TBP incubator (MPM Instruments, Bernareggio, Italy). 150 μL aliquots were sampled from release media at defined dialysis times (0.05, 2, 4, 6, 8, 10, 24.5 and 49 h) and each time replaced with an equal volume of fresh release medium. All aliquots from dialysis experiments were then submitted to HPLC-UV analysis and injected (*n* = 1; injection volume: 20 µL) 10 min after collection to minimize the degradation of analytes outside the dialysis settings.

The degradation of PTX_2_S was investigated in the same timeframe and redox conditions by HPLC-UV analysis on five samples of PTX_2_S (25 µg/mL), prepared by dilution of a 10 mg/mL stock solution in DMSO with release media A–E. Samples were incubated at 37 °C inside the HPLC autosampler for the whole duration of the dialysis experiment (49 h). The main degradation products of PTX_2_S were identified by inspection of UV profiles from PDA spectra, analysis of pure standards and comparison with literature studies. The kinetics of PTX_2_S conversion to PTX was evaluated by plotting the percent peak area of both compounds as a function of time. The main degradation products of Pba were also investigated by liquid chromatography coupled to high-resolution mass spectrometry (LC–HRMS) using a Xevo G2-XS QTof mass spectrometer connected to a Acquity UPLC H-Class system (Waters, Sesto San Giovanni, Italy).

HPLC-UV analysis was performed on a Nexera XR UHPLC system (Shimadzu, Milan, Italy) equipped with a LC-40D XR pump, a SIL-40C XR autosampler, a DGU-405 degassing unit, a CTO-40S column oven, a SPD-M40 PDA detector, and a Kinetex C18 column (5 µm, 100 Å, 150 × 4.6 mm; Phenomenex, Castel Maggiore, Italy). Mobile phases A (TFA 0.1% in water, *v*/*v*) and B (TFA 0.1% in ACN, *v*/*v*) were used to develop a gradient at a constant flow rate of 1.25 mL/min: 0 min, 33% B; 5 min, 83% B; 7 min, 83% B; 7.5 min, 33% B; 12 min, 33% B. Two detection wavelengths were extracted from PDA data (700–200 nm) for the simultaneous monitoring of PTX derivatives (228 nm) and Pba (413 nm). Column oven and autosampler temperatures were set to 40 °C and 37 °C, respectively.

The quantification of released PTX and Pba was performed by single-point calibration with two standard samples of PTX (30 µg/mL) and Pba (15 µg/mL), prepared by dilution of 10 mg/mL and 1.5 mg/mL stock solutions in DMSO with release medium A, respectively. Kinetics profiles for PTX and Pba were derived from the fractions of released compounds relative to the total content of NP samples (PTX: 386.5 µg; Pba: 106.2 µg), as determined from the cumulative peak area of all significant precursors and derivatives (Tables S1 and S2) under the assumption that their UV absorption can be approximated to that of PTX (*t*_R_ = 4.7 min) and Pba (*t*_R_ = 8.2 min).

### 2.7. Binding of PTX Conjugates to HSA

#### 2.7.1. Reduction in HSA

A 150 µM stock solution of HSA and a 14.85 mM stock solution of dl-dithiothreitol (DTT) were prepared in potassium phosphate buffer (50 mM, pH 7.4). Reversibly oxidized Cys34 was reduced by incubating HSA with DTT (final concentration of HSA and DTT: 148.5 µM) for 1 h at room temperature. The resulting solution was ultrafiltered with Amicon Ultra tube (0.5 mL, MWCO: 10 kDa). 20 cycles of centrifugation (6 min, 10,000× *g*, room temperature) were used to remove DTT; after each cycle, 350 µL of fresh potassium phosphate buffer (50 mM, pH 7.4) were added to the reservoir containing the concentrated HSA solution. The molar concentration of HSA was assessed by spectrophotometric analysis. Percentage of free Cys34 before and after the reduction was assessed by liquid chromatography–mass spectrometry (LC–MS) analysis applying a previously published procedure [38].

#### 2.7.2. Reaction of HSA with **MAL** and **BOC**

HSA was incubated for 30 min at 37 °C in the presence of excess of **MAL** or **BOC** (molar ratio 10:1) in potassium phosphate buffer (50 mM, pH 7.4) under gentle shaking (400 rpm) using a Thermomixer Comfort (Eppendorf). Mixtures were then diluted with potassium phosphate buffer 50 mM, pH 7.4 at the final HSA concentration of 7.5 µM, and the yield of reaction was assessed by HPLC–UV analysis. The time- and concentration-dependent ability of **MAL** to bind HSA was assessed by incubating HSA (25 µM) with **MAL** at different **MAL**/HSA molar ratios (5, 2.5 and 1) and monitoring the yield of reaction at different incubation times (0, 30, 60, 120, 180, 240, 300 min) by HPLC–UV analysis. Analyses were carried out on a HPLC Jasco PU-980 equipped with a Jasco MD-910 multiwavelength detector and using a C4 column (150 × 2.0 mm, 5 µm, 300 Å; Phenomenex Jupiter). A gradient was developed with mobile phases A (water/ACN/TFA, 70:30:0.3, *v*/*v/v*) and B (water/ACN/TFA, 30:70:0.3, *v*/*v*/*v*): 0 min, 10% B; 10 min, 65% B; 11 min, 80% B; 13 min, 80% B. Column was equilibrated with the starting conditions for 10 min before the next injection. The flow rate was 0.4 mL/min and the injection volume was 20 µL. Detection wavelength was set at 230 nm. Analyses were performed in duplicate. The yield of reaction was calculated from the decrement of the peak area for HSA (*t*_R_ = 7.7 min), corrected by the relative abundance of HSA with Cys34 in reduced form as assessed by LC–MS analysis.

### 2.8. In Vitro Studies on Breast Cancer Cell Monolayers

#### 2.8.1. Combination Therapy Efficacy and CI Analysis

For combination therapy experiments, cells (8 × 10^3^ cells/well for MDA-MB-231 and MCF7, 3.5 × 10^3^ cells/well 4T1) were seeded in 96-well plates, and after 24 h of growth the medium was replaced with fresh one containing **MAL**-PTX_2_S@Pba, **BOC**-PTX_2_S@Pba, nPTX_2_S and Pba. Cells were incubated with drugs/nanoparticles for 24 h and cell viability was measured with MTS assay after 24 h or 48 h during which cells were kept in drug-free medium (dark cytotoxicity; time points 24 h and 48 h). For photo-toxicity experiments (PDT in vitro), cells were seeded and treated as described above, and upon 24h incubation, cells were washed twice with PBS Ca^2+^ and Mg^2+^ and irradiated with red light (600–750 nm) emitted by a PDT1200 Waldmann lamp with a total fluence of 1 J/cm^2^. The power density was 20 mW/cm^2^ as measured with the radiometer PDT 1200L (Waldmann, Baden-Württemberg, Germany). After irradiation, and upon replacement of PBS with fresh medium, cells were incubated in the dark for 24 or 48 h prior viability assessment by MTS assay (phototoxicity; time points: 24 h or 48h). For MTS assay, the medium was replaced with 100 μL of serum-free medium and 20 μL of the CellTiter 96^®^ reagent. After 1 h, the absorbance at 492 nm was measured with a Multiskan Go (Thermo Fischer Scientific, Waltham, MA, USA) plate reader and cell viability was expressed as a function of absorbance relative to that of control cells (considered as 100% viability). In order to determine if the use of dual-loaded nanoparticle resulted in a synergistic effect, the combination index (CI) values were calculated using the CompuSyn software (ComboSyn Inc., Paramus, NJ, USA) based on the Chou and Talalay method along with Drug Reduction Index values [39].

#### 2.8.2. Intracellular Uptake of Pba

The internalization rate of Pba either as free or loaded in nanoparticles was measured by flow cytometry. Breast cells (6 × 10^4^ cells/well) were grown in 24-well plates for 24 h and incubated for 1 or 4 h with 0.25 μM Pba (free drug or NPs loaded). At the end of the incubation time, cells were washed twice with Versene solution, detached from the plates with trypsin that was neutralized by the addition of FBS. Cells were centrifuged and resuspended in Versene before measuring Pba fluorescence using a BD Fortessa^TM^ X-20 flow cytometer (Becton Dickinson, San Jose, CA, USA). A red laser (640 nm) was used to excite the PS and its fluorescence was detected at wavelengths >670 nm (APC channel). For each sample, 10^4^ events were acquired and analyzed using the FACSDiva and FlowJo softwares.

#### 2.8.3. Scratch Wound Assay

4T1 cells were seeded in a 24-well plate at a density of 1 × 10^5^ cells/ well and grown for another 24 h until reaching 80% confluence. Cells were treated with Pba, nPTX_2_S, **MAL**-PTX_2_S@Pba and **BOC**-PTX_2_S@Pba nanoparticles ([Pba] = 0.16 μM; [PTX] = 0.4 μM), incubated in the dark for 4h and then irradiated with red light at a fluence of 1.2 J/cm^2^. Afterwards, a denuded area was created across the diameter of the dish by a yellow tip. The cells were then washed with PBS and further incubated in complete medium. At different times after scratch, pictures were taken with an Epifluorescence microscope Leica DMI6000B (Leica Microsystem, Heidelberg, Germany) at a magnification of 10× to evaluate the migration distance. The area of wound closure was calculated as percentage of the initial wound area using the Leica Application Suite X (LAS X) 3.5.5 software.

### 2.9. In Vitro Studies on Breast Cancer 3D Breast Cancer Tumor Models

#### 2.9.1. 3D-Models Generation

Spheroids of MDA-MB-231 and 4T1 cells were generated into 96 wells Ultra Low Attachment (ULA) plates (Nunclon^TM^ Sphera^TM^ U-shaped bottom microplates, Thermo Fischer Scientific, Waltham, MA, USA). Briefly, 4 × 10^3^ MDA-MB-231 cells/well were seeded in DMEM supplemented with 10% FBS and 25 μL/mL of collagen I (BD Biosciences, San Jose, CA, USA) while 2 × 10^3^ 4T1 cells/well were seeded in DMEM supplemented exclusively with 10% FBS. Immediately after seeding, cells were centrifuged for 3 min at 100× *g* to promote cell aggregation and allowed to grow in the incubator for 3 days at 37 °C under 5% CO_2_ before their use in the following experiments.

#### 2.9.2. Cytotoxicity and Phototoxicity Experiments

Three-days-old spheroids of MDA-MB-231 or 4T1 cells were incubated with 100 μL of fresh medium containing 10% FBS and increasing concentration of **MAL**-PTX_2_S@Pba, **BOC**-PTX_2_S@Pba, nPTX_2_S and Pba. After 24 h of incubation, if cell irradiation was foreseen, spheroids were washed with PBS containing Ca^2+^ and Mg^2+^ and irradiated in PBS with a red light fluence of 1 J/cm^2^ (power density 20 mW/cm^2^). Immediately after irradiation, spheroids were stored in the incubator after the replacement of PBS with fresh medium. After additional 24 or 48 h, cell viability was measured using the CellTiter-Glo^®^ 3D Cell Viability Assay. Briefly, 100 μL of cell medium were left in each spheroid well and 100 μL of CellTiter-Glo^®^ 3D reagent (Promega Italia, Milan, Italy) were added; the well was incubated at room temperature for 30 min before transferring the 200 μL of sample/wells in a blank 96/well (Perkin Elmer, Waltham, MA, USA). Luminescence was measured with a Perkin Elmer VICTOR^TM^X3 instrument.

#### 2.9.3. LIVE/DEAD Assay

The cell death extent in spheroids treated with **MAL**-PTX_2_S@Pba, **BOC**-PTX_2_S@Pba, nPTX_2_S and Pba for 24 h and exposed to 1 J/cm^2^ red light was assessed after staining with the LIVE/DEAD^®^ viability/cytotoxicity assay kit (Invitrogen, Milan, Italy). Twenty-four or forty-height hours after irradiation, spheroids were rinsed with PBS and then 150 μL of PBS solution containing 1 μM calcein AM and 4 μM ethidium homodimer-1 (EthD-1) was added to each well for the simultaneous determination of live (calcein positive) and dead (ethidium positive) cells. After 30 min of incubation at 37 °C, spheroids were washed twice with PBS and analyzed with a fluorescence microscope (DMI4000, Leica Microsystems). Acquired images were analyzed by quantifying fluorescence intensity (green and red fluorescence for live and dead cells quantification within the spheroid area, respectively) by using ImageJ software.

#### 2.9.4. Uptake Experiments

The localization of Pba delivered with the three formulations was evaluated by confocal microscopy in MDA-MB-231 and 4T1 spheroids exposed to the drug for 24 h. Afterwards, spheroids were transferred from 96-well plates to 35 mm μ-Dish, washed twice with PBS and observed on Leica SP5 microscope. Images were acquired from the top to the equatorial plane of the spheroid in about 50 different focal planes. Images were elaborated with the LAS X software (Leica) to create the images with the super-imposition of all the acquired focal planes.

### 2.10. Animal Studies

Female 6-week-old BALB/c Ola Hsd mice were purchased from Envigo Srl (Udine, Italy) and maintained in a conventional animal house for 2 weeks. All procedures were performed in accordance with the guidelines on Care and Use of Laboratory Animals provided by the Italian Ministry of Health and approved by the Institutional Animal Care Committee of the University of Udine (Italian Ministry of Health approval number: 423/2019- PR). Every effort was undertaken to avoid unnecessary pain to the animals.

#### 2.10.1. Pba Tumor Biodistribution in Mice Bearing 4T1 Tumor

To assess the best timing for performing the PDT treatment, the in vivo tumor accumulation of **MAL**-PTX_2_S@Pba and **BOC**-PTX_2_S@Pba nanoparticles was evaluated by exploiting the fluorescence emission of Pba. Female 6-week-old BALB/c OlaHsd mice (18-20 g weight) were subcutaneously injected with 1 × 10^6^ 4T1 breast cancer cells in the upper flank. Pba (2.34 mg/Kg mouse) and the corresponding amounts of **MAL**-PTX_2_S@Pba and **BOC**-PTX_2_S@Pba were dissolved in 100 μL of injectable water and administered intravenously (i.v.) when tumors had reached a volume of 400 mm^3^. Animals were kept in the dark and sacrificed (three mice for each time point) at different time points (1, 3, 6 and 24 h) after drug administration. Tumors were carefully excised and homogenized in MeOH:DMSO (4:1 *v*/*v*) and the amount of Pba in the tumor was determined by the method of Villanueva and Jori [40]. The homogenates were centrifuged and the amount of Pba in the supernatant was measured by fluorescence (Ex. 420 nm, Em. 450 to 750 nm). No interference was detected from other compounds present in the extracts. The amount of Pba in the tumor was determined using a calibration curve obtained by plotting the fluorescence intensity against the Pba concentration using a standard Pba solution. The value was expressed in μg/mg tissue. The curve was linear in the range from 0 to 2 μg/100 mg tissue with a correlation coefficient r^2^ = 0.9994. The assay was highly reproducible with an error rate of <8%.

#### 2.10.2. Antitumor Activity of Combined Therapy

Female 6-week-old BALB/c OlaHsd mice (18–20 g weight) were subcutaneously injected with 1 × 10^6^ 4T1 breast cancer cells in the upper flank. After 1 week, the tumors had reached a size of 6–8 mm (largest diameter), and the mice were randomly divided into five groups of 7 mice each and injected intravenously with 100 μL of each formulation corresponding to 8.16 mg/Kg of PTX and 2.34 mg/Kg of Pba. Three hours post-injection, when Pba was present in similar amounts in the tumor, mice were anesthetized with ketamine + xylazine (40 mg/kg + 20 mg/kg; i.p.), shaved in the tumor area, and irradiated with a BWN-660-60E laser (B&WTEK, Inc., Newark, DE, USA) at 660 ± 5 nm, fluence of 193 J/cm^2^. The mice were examined every 2 days for weight changes, the occurrence of side effects, or signs of nausea. Tumor size was measured every 2–3 days using calipers. Tumor mass (mg) was calculated assuming a tumor density of 1 and a tumor volume of π/6 × a2 × b, where a and b are the shorter and larger axes (cm), respectively. After 14 days of treatment, all mice were sacrificed. Tumors were measured and weighed. Lungs were harvested for the number and weight of metastases by stereomicroscope.

#### 2.10.3. Statistical Analysis

Analyses of primary tumor growth and metastasis were performed using Graph Pad PRISM software (GraphPad; San Diego, CA, USA). All results were expressed as the mean ± SE (standard error). Two-way ANOVA followed by Tukey’s multiple comparisons test was used to determine statistical probabilities in the tumor growth whereas by Dunnet multiple comparison test for the number of metastases. Results were statistically significant at *p*-values < 0.05.

## 3. Results

### 3.1. Synthesis and characterization of PTX-SS-PEG-**MAL** (**MAL**), PTX-SS-PEG-**BOC** (**BOC**) and PTX_2_S

**Figure 1:**
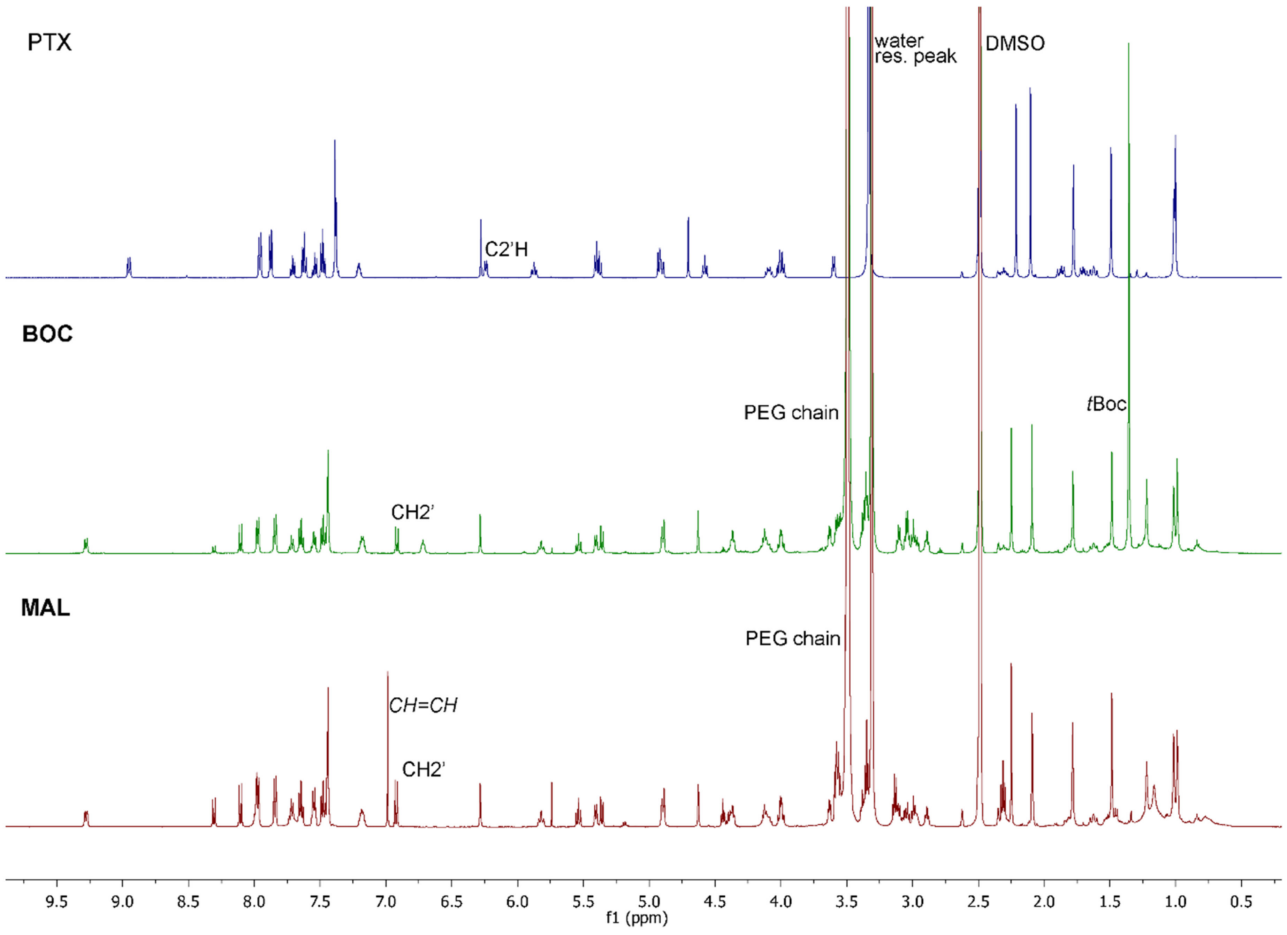
^1^HNMR comparison between PTX (dark blue), **BOC** (green), **MAL** (dark red). Samples were dissolved in DMSO, and spectra recorded on a 500 MHz Varian spectrometer.

To achieve our goal, the amphiphilic HSA-binding **MAL** derivative was synthesized along with its non-HSA binding analog, namely **BOC** derivative, via a four-steps procedure starting from commercially available PTX (**1**) (Scheme 1a). Firstly, the C2’-OH of the PTX side chain was reacted with 4-nitro-phenyl chloroformate to afford PTX-PNP (**2**) that upon reaction with 2-hydroxyethyl disulfide in THF/DCM mixed solvents, provided derivative PTX-SS-OH (**3**) in 45% isolated yield over two steps. Compound **3** was then activated with 4-nitro-phenyl chloroformate as reported for the previous step to afford derivative **4**, whose structure was confirmed by ^1^H-NMR spectroscopy (Figure S1). Derivative **4** was subsequently reacted with NH_3_^+^-PEG_45_-**BOC** or NH_3_^+^-PEG_45_-**MAL** to provide derivatives **BOC** (**5**) and **MAL** (**6**), respectively, in good, isolated yields. Compounds **MAL** and **BOC** were purified by dialysis against ultrapure H_2_O, and their structures were confirmed by ^1^H-NMR analysis (Figure 1).

As recently reported by us and other authors [30,41], the PTX_2_S dimer was obtained through the straightforward reaction between PTX and 2,2’-thiodiacetic acid under EDC/DMAP coupling conditions (Scheme 1b).

Figure 1 reports the comparison between the ^1^HNMR spectra of PTX, **MAL** and **BOC**, which clearly shows the downfield shift of the C2’H signal from 6.24 ppm to 6.93 ppm and the presence of the CH_2_ signals of the linker chain in the 2.84–3.20 region. Both **MAL** and **BOC** spectra clearly show the presence of the PEG chain centered at 3.49 ppm, and signals of the maleimide ring and *^t^*BOC group are clearly visible at 6.99 ppm and 1.35 ppm for derivative **MAL** and **BOC**, respectively (see also supporting information, Figures S2 and S3).

### 3.2. Preparation, Stability and Morphological Characterization of **MAL**-PTX_2_S@Pba, **BOC**-PTX_2_S@Pba, nPTX_2_S Nanoparticles

**Figure 2:**
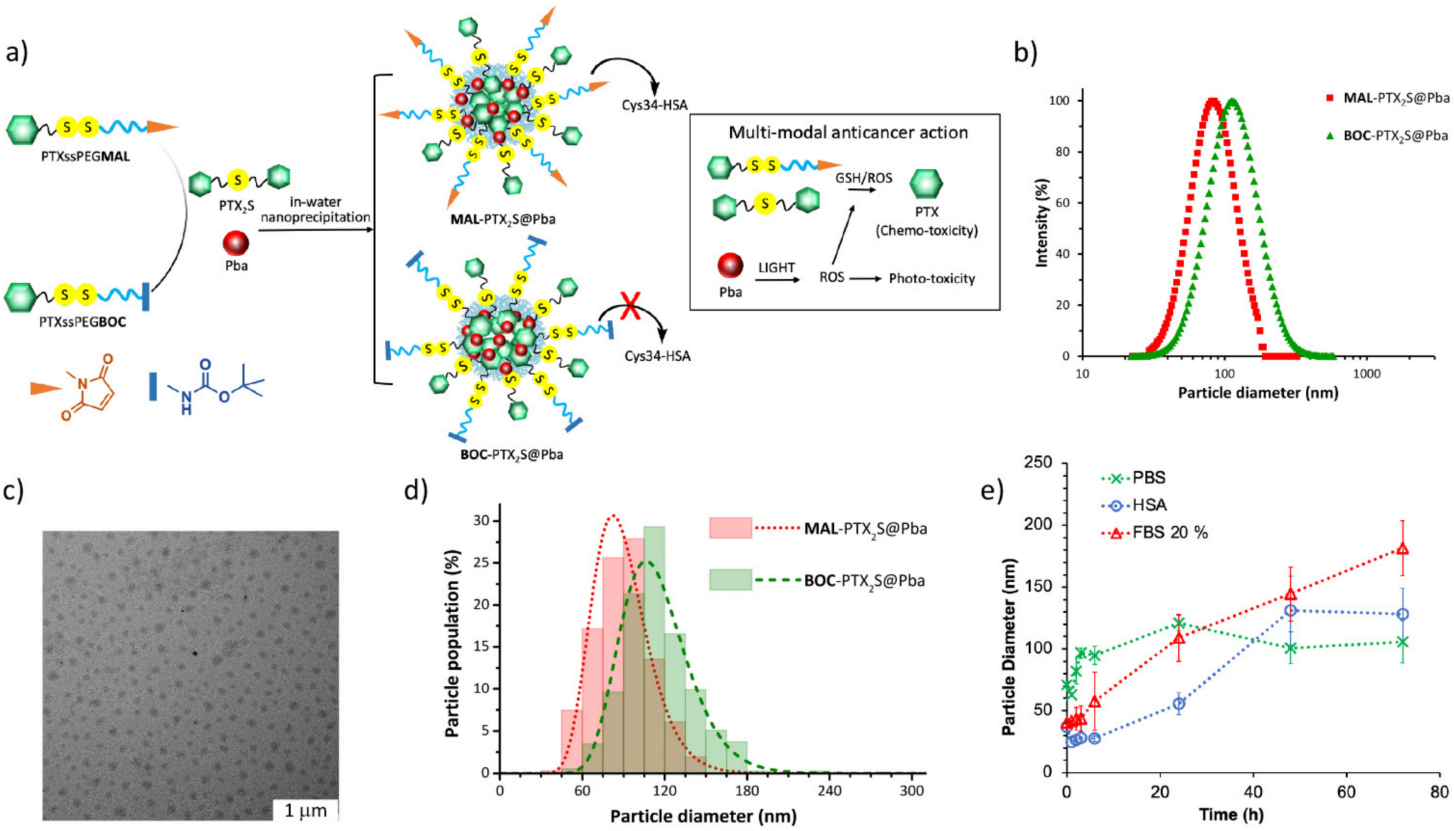
(**a**) Schematic representation of the structure and mode of action of **MAL**-PTX_2_S@Pba and **BOC**-PTX_2_S@Pba NPs. (**b**) Representative DLS measurements on **MAL**-PTX_2_S@Pba (red squares) and **BOC**-PTX_2_S@Pba (green triangles) NPs. (**c**) TEM analysis of **MAL**-PTX_2_S@Pba (scale bar 1μm). (**d**) Particle size distributions of **MAL**-PTX_2_S@Pba (red dotted line) and **BOC**-PTX_2_S@Pba (green dashed line) NPs, as determined by TEM analysis. (**e**) Particle size stability of **MAL**-PTX_2_S@Pba NPs in PBS pH 7.4 (green dotted line), FBS 20% in PBS pH 7.4, *v*/*v* (red dotted line), and HSA 35 mg/mL in PBS pH 7.4, *v*/*v* (blue dotted line), as determined by DLS analysis.

Based on our previous results [30], demonstrating that the thioether dimer of PTX (PTX_2_S) is able to spontaneously form nanoparticles incorporating the hydrophobic photosensitizer Pba, we envisioned to take advantage of this feature to fine tune the preparation of novel HSA-binding nanoparticles. Indeed, **MAL**-PTX_2_S@Pba nanoparticles were obtained by the nanoprecipitation method, involving the simoultaneous and slow injection of DMSO solutions of **MAL**, PTX_2_S and Pba into water (Figure 2a). An optimization study allowed to define the best **MAL**/PTX_2_S/Pba ratio (*w*/*w*%) for obtaining stable and reproducible nanoparticles. In particular, we observed that, by increasing the amount of PTX_2_S and lowering the Pba *w*/*w*% content, smaller and more monodisperse NPs were formed (Figure S4), confirming the extraordinary aggregating properties of the PTX thioether dimer. Based on this study we obtained reproducible nanoparticles with an average hydrodynamic diameter of 82 ± 1 nm, a PDI of 0.17 ± 0.015 (Figure 2b), an almost neutral ζ-potential (2 mV), and with a final **MAL**/PTX_2_S/Pba ratio of 56%:32%:12% *w*/*w*/*w*, that translates into a PTX equivalent/Pba ratio of 2.5, e.g., 0.52 μmol eq. of PTX and 0.21 μmol of Pba per mg of NPs.

It is worth noticing that our approach relies on the use of two different PTX prodrugs, endowing several advantages including the formation of more stable nanoparticles bestowed with a PEGylated shell that should reduce in vivo nanoparticles opsonization. Furthermore, a fine tuning of PTX equivalents as respect to Pba content is allowed, since the dimer provides 2 PTX equivalents, while the **MAL** conjugate contributes with one PTX equivalent. Most importantly, this procedure takes advantage of two different bioresponsive linkers within the same nanoparticle, capable of releasing of PTX. Most tumor cells are characterized by an imbalanced oxidation state due to ROS and GSH overproduction [42]. These features can be cunningly exploited to promote the smart release of drugs preferentially at the tumor site. Indeed, the thioether (-S-) and disulfide (-SS-) bridges are amongst the most used and effective redox linkers; specifically, the thioether bond is more sensitive to ROS, whereas the disulfide bridge is more sensitive to GSH [18,43] Therefore, having both these linkers within the same nanocarrier could promote a more effective and controlled drug release by exploiting the different features of the tumor environment, as well as the PDT process.

To better discriminate the ability of **MAL**-PTX_2_S@Pba NPs to interact with HSA in vitro and in vivo, we also prepared the corresponding non-HSA binding **BOC**-PTX_2_S@Pba NPs as negative control. **BOC**-PTX_2_S@Pba NPs were prepared based on the same procedure and with comparable amounts of PTX prodrugs and Pba, affording monodisperse spherical particles of 113 nm ± 1 nm with a PDI of 0.13 ± 0.012 (Figure 2b). Particle size analysis performed on TEM images (Figure 2c and Figure S5) confirmed the results of DLS measurements: number-based particle size histograms were fitted to log-normal distribution functions (Figure 2d), yielding a mean diameter of 89 nm (*n* = 362, PDI = 0.06) for **MAL**-PTX_2_S@Pba NPs and 114 nm (*n* = 375, PDI = 0.05) for **BOC**-PTX_2_S@Pba NPs.

We next investigated the stability of **MAL**-PTX_2_S@Pba NPs under different conditions of medium. Stability was evaluated at 37 °C for 72 h in PBS pH 7.4, FBS 20% in PBS pH 7.4, *v*/*v* and HSA 35mg/mL in PBS pH 7.4, *v*/*v* (Figure 2e). Our results indicate that, in PBS, **MAL**-PTX_2_S@Pba NPs are stable within the observation timeframe with no significant changes in the hydrodynamic diameter (Figure 2e, green dotted line). Interestingly, the presence of 20% FBS (Figure 2e, red dotted line) induced a gradual size increase, likely because of an interaction with serum proteins. However, nanoparticle sizes remained within an acceptable range (<200 nm) and no visible aggregation/precipitation phenomena were observed. Remarkably, stability studies performed in the presence of HSA (35 mg/mL), i.e., the average HSA plasma concentration, showed that particles are very stable up to 24 h and no aggregation was observed up to 72 h despite a larger particles’ size was achieved, e.g., 138 nm (Figure 2e, blue dotted line). Conversely, the same experiments performed on **BOC**-PTX_2_S@Pba NPs showed that these particles are less stable and start to aggregate already after 2 hours (Figure S6). Overall, these data confirm the key role of **MAL** decoration to provide stable and possibly long-circulating nanoparticles.

### 3.3. PTX and Pba Release from **MAL**-NPs

A meaningful description of drug release from **MAL**-PTX_2_S@Pba NPs poses several non-trivial experimental challenges. The extremely poor solubility of PTX in water (~0.3 μg/mL) can significantly hamper the quality of release experiments [18]; dialysis experiments were therefore carried out using release media based on PBS pH 7.4 and ethanol (70:30, *v/v*) to improve the solubility of PTX. The two PTX prodrugs (**MAL** and PTX_2_S) should have different mechanisms of conversion into the active drug, possibly leading to complex kinetics of PTX release from NPs. Moreover, both PTX and Pba undergo significant degradation in slightly alkaline aqueous environments at 37 °C, which are typical near-physiological settings of in vitro release experiments. HPLC-UV and LC-MS analysis allowed us to detect and identify several derivatives formed by epimerization [44] and hydrolysis [45] of PTX, oxidation of Pba [46], and conversion of **MAL** and PTX_2_S to PTX (Tables S1 and S2). Release kinetic profiles were thus derived from the cumulative peak area of all the significant PTX and Pba derivatives detected over time, aiming at simplifying the interpretation of the multiple kinetics of reactions occurring simultaneously during dialysis.

**Figure 3:**
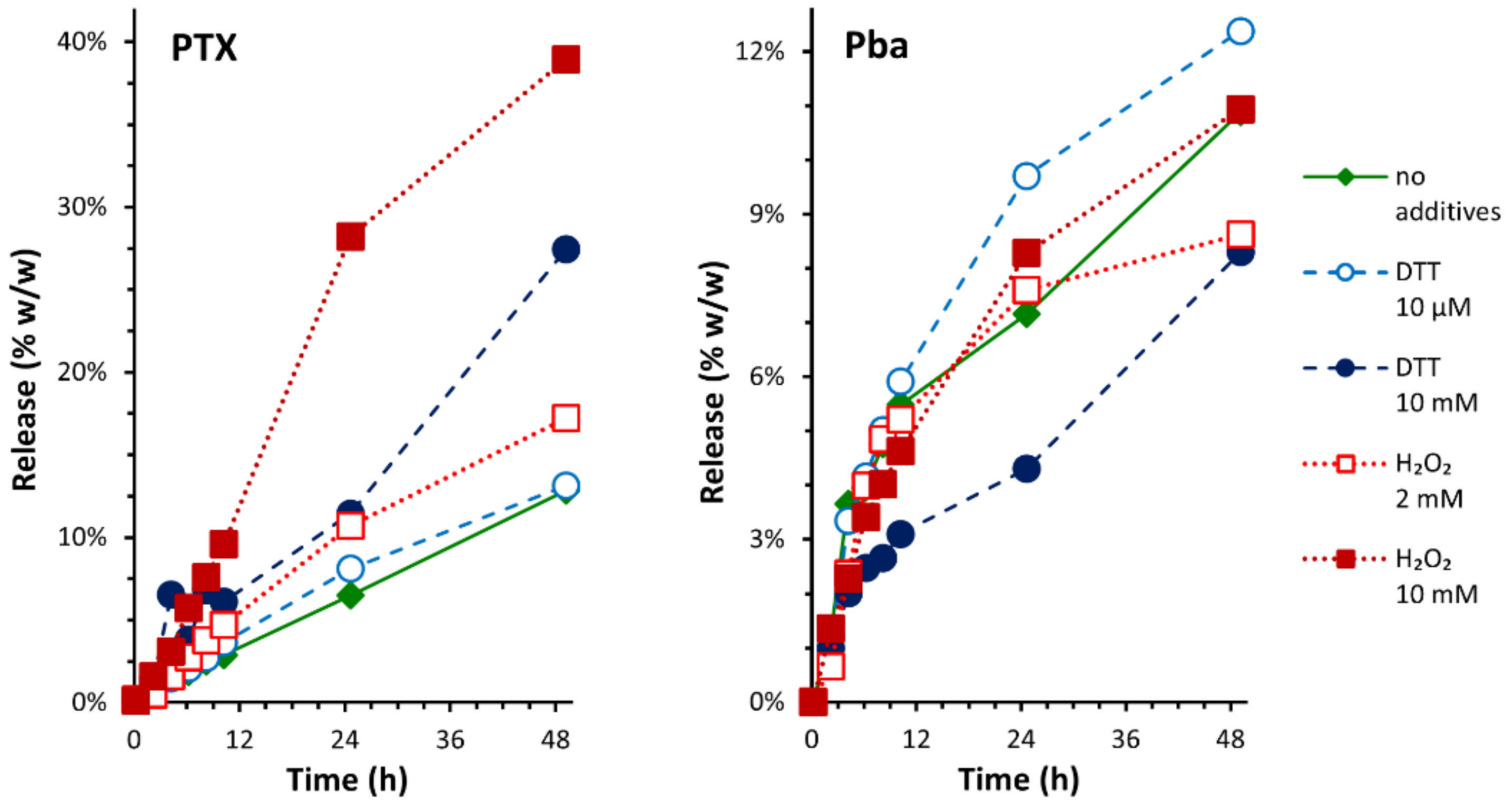
Release profiles of PTX and Pba from **MAL**-PTX_2_S@Pba NPs in different redox conditions, as determined by equilibrium dialysis and HPLC–UV analysis. Diamonds: redox-neutral; empty circles: mildly reducing; filled circles: strongly reducing; empty squares: mildly oxidizing; filled squares: strongly oxidizing. Redox conditions are described in detail in the Experimental section (Section 2.5).

In this framework, the observed release profiles of PTX and Pba from **MAL**-PTX_2_S@Pba NPs (Figure 3, Table S3) suggest that the release of PTX is faster in strongly oxidizing conditions (H_2_O_2_ 10 mM), while the release of Pba appears to be less influenced by the redox environment. HPLC-UV data on the stability of PTX_2_S (Figure S7) show that strongly oxidizing conditions lead to a faster rate of conversion to PTX, which in turn hydrolyzes faster than in reducing conditions. On the other hand, the delayed increase in PTX release from NPs in strongly reducing conditions (DTT 10 mM) might be due to conversion of **MAL** to PTX, as suggested by the simultaneous appearance of a new derivative which is not observed during the stability studies on PTX_2_S (Table S1, Figure S8). Overall, these results are consistent with a different mechanism of conversion for the two PTX prodrugs (favored by oxidizing conditions for PTX_2_S, by reducing conditions for **MAL**) and with the proposed redox-selective release mechanism of PTX from **MAL**-PTX_2_S@Pba NPs.

### 3.4. Albumin Binding Properties of PTX Prodrugs

**Figure 4:**
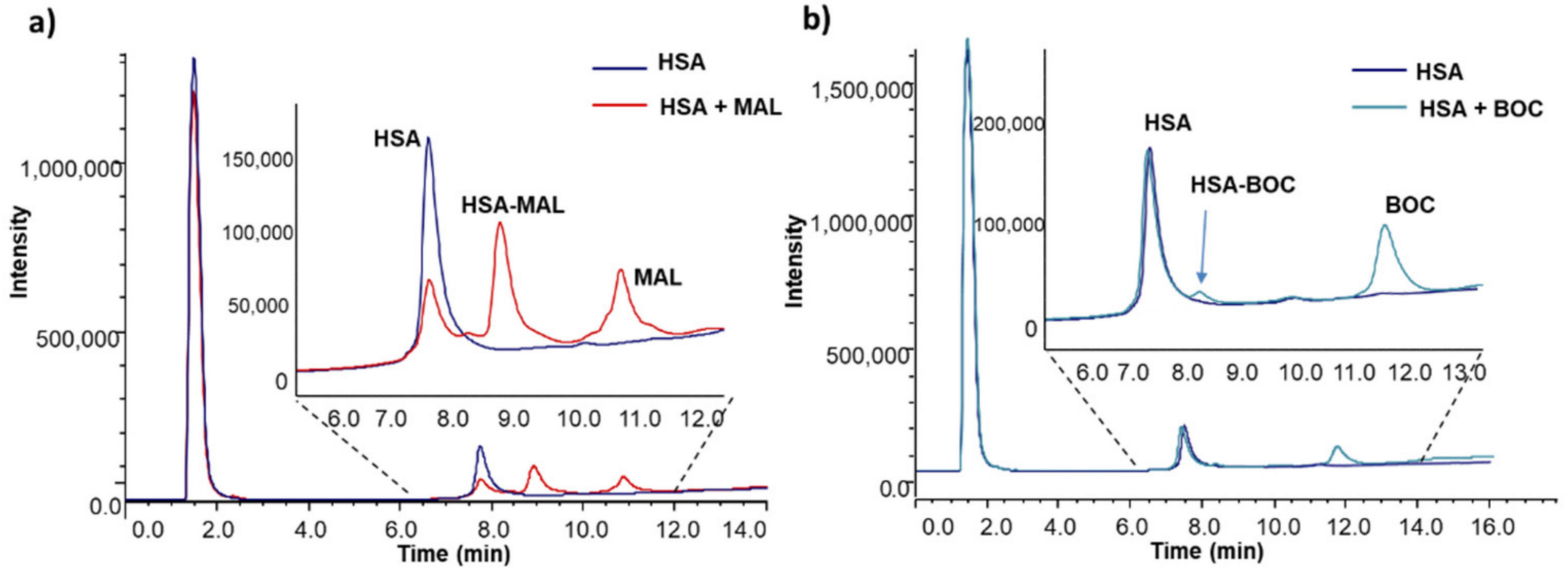
LC-MS analysis of adduct formation between HSA and **MAL** (**a**) or **BOC** (**b**). HSA concentration was 25 µM while **MAL** and **BOC** concentration was 250 µM, reaction buffer was potassium phosphate 50 mM, pH 7.4, incubation time was 30 min, and reaction temperature was 37 °C. (**a**) Rt HSA = 7.7 min; Rt HSA-**MAL** adduct = 8.3 min; Rt **MAL** = 10.9 min. (**b**) Rt HSA = 7.7 min; Rt HSA-**BOC** adduct = 8.2 min; Rt **BOC** = 11.8 min.

The ability of **MAL** vs. **BOC** to bind HSA was assessed in vitro by incubating commercial HSA with the two prodrugs in potassium phosphate 50 mM, pH 7.4 for 30 min at 37 °C; since in commercial samples Cys34 is partially involved in the formation of a disulfide bridge with another Cys residue, prior to reaction with **MAL** and/or **BOC**, HSA was pretreated with DTT to reverse disulfide bridges to free thiols. Amount of free Cys34 upon DTT treatment was 64.8%, as assessed by mass spectrometry analysis. In agreement with the rationale of the study **MAL** effectively binds HSA forming a stable adduct with a reaction yield of 94% when a 10-fold molar excess of prodrug was used (Figure 4a). In the same experimental conditions, the non-binding counterpart **BOC** very weakly reacts with HSA with a reaction yield of only 4.6% (Figure 4b).

As expected, the formation of the **MAL**-HSA adducts proved to be dose and time-dependent (Figure S9). Reaction was fast and, independently from the molar excess of **MAL** over HSA, maximum yield was reached in 1h. Yield of reaction was dose-dependent, increasing with increased **MAL**/HSA molar ratio (i.e., 24%, 68%, 94% when molar ratio of 1, 2.5 and 5 were used, respectively).

### 3.5. In Vitro Combination Therapy with **MAL**-PTX_2_S@Pba

**Figure 5:**
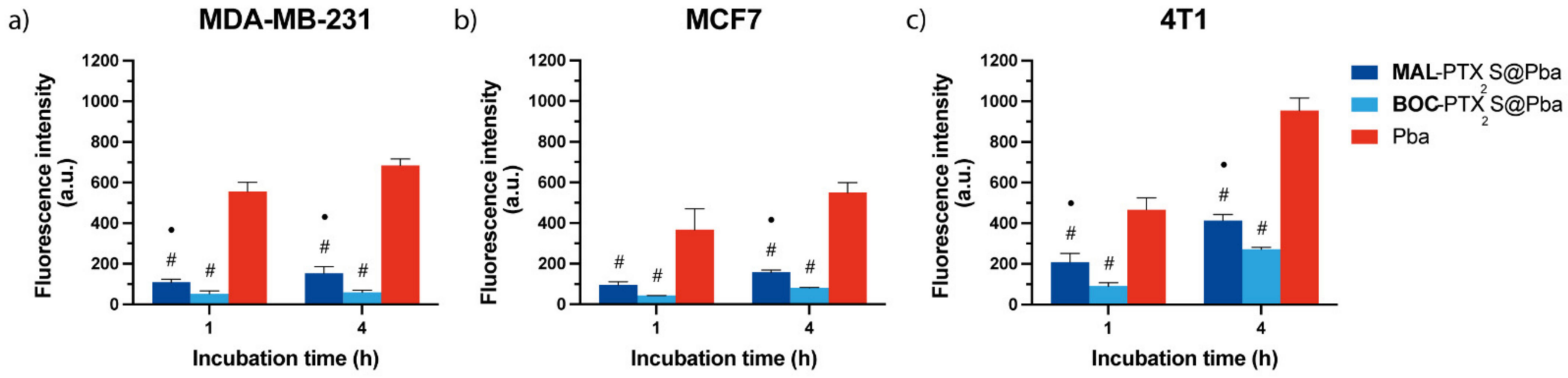
In vitro uptake of the different Pba formulations in MDA-MB-231 (**a**), MCF7 (**b**) and 4T1 (**c**) cells cultured as monolayers. Cells were incubated for 1 or 4h with 0.25 μM of Pba dissolved in the standard solvent or loaded in **MAL**-PTX_2_S@Pba or in **BOC**-PTX_2_S@Pba, and the uptake of Pba was measured by flow cytometry at the end of incubation times. Data are expressed as mean percentage ± SD of least three independent experiments, carried out in triplicate. Statistical significance was calculated applying the two-way ANOVA with Bonferroni’s correction: ● significantly different from **BOC**-PTX_2_S@Pba; # significantly different from Pba.

Based on the proved ability of **MAL** derivative to selectively bind HSA as respect to its non-binding counterpart, e.g., **BOC**, (Figure 4) we assessed in in vitro cell monolayers obtained from three different breast cancer lines (e.g., human MDA-MB-231, MCF7 and mouse 4T1), if the presence of the albumin-binding moiety on **MAL**-PTX_2_S@Pba nanoparticles could effectively enhance their intracellular uptake. Flow cytometry data based on Pba red fluorescence measured after 1 or 4h of cell incubation with the different formulations (Figure 5) indicated that **MAL**-PTX_2_S@Pba significantly increase Pba uptake in all cell lines with respect to the corresponding **BOC**-PTX_2_S@Pba nanoparticles. Indeed, the use of **MAL**-decorated NPs induced a two-fold increase in PS uptake with respect to **BOC**-NPs, very likely due to the occurrence of albumin-mediated endocytosis processes, as known for **MAL**-nanoparticles/transported drugs [18]. Of note, when the same experiment was performed in non-malignant breast epithelial MCF10A cells, no significant differences were observed in Pba uptake between **MAL**-PTX_2_S@Pba and **BOC**-PTX_2_S@Pba NPs (Figure S10), thus confirming, at least in vitro, the capability of HSA-binding nanocarriers to increase drug selective internalization exclusively in cancer cells.

Despite the higher Pba uptake promoted by **MAL**-decorated NPs, the highest extent of Pba intracellular internalization was achieved when the PS was delivered in standard solvent, resulting 5, 4 and 3 times higher with respect to **MAL**-PTX_2_S@Pba in MDA-MB-231, MCF7 and 4T1 cells, respectively. These results are not surprising and in line with previous reports indicating that nanoparticles PEGylation reduces their non-specific protein adsorption, thus resulting in a decreased uptake in cancer cell lines in vitro [47]. Overall, it is important to highlight that the Pba endocytosis process is not restricted to malignant cells as observed by us (Figure S10) and other researchers [48], while the albumin-based endocytic approach exploited by **MAL**-PTX_2_S@Pba nanoparticles could significantly enhance tumor tissues’ preferential internalization.

Based on previous evidence on the capability of PTX_2_S/Pba untargeted NPs to be efficiently and specifically activated in the reductive environment of cancer cells, in turn promoting micelle disassembly and Pba release [30], we investigated the antitumor efficacy of our chemotherapy/PDT approach using PEGylated and HSA-targeted nanoparticles containing Pba and two different PTX prodrugs, i.e., PTX_2_S and **MAL**. The anticancer efficacy of combination therapy using **MAL**-PTX_2_S@Pba was compared with **BOC**-PTX_2_S@Pba, while free Pba and **n**PTX_2_S were used as control references.

**Figure 6:**
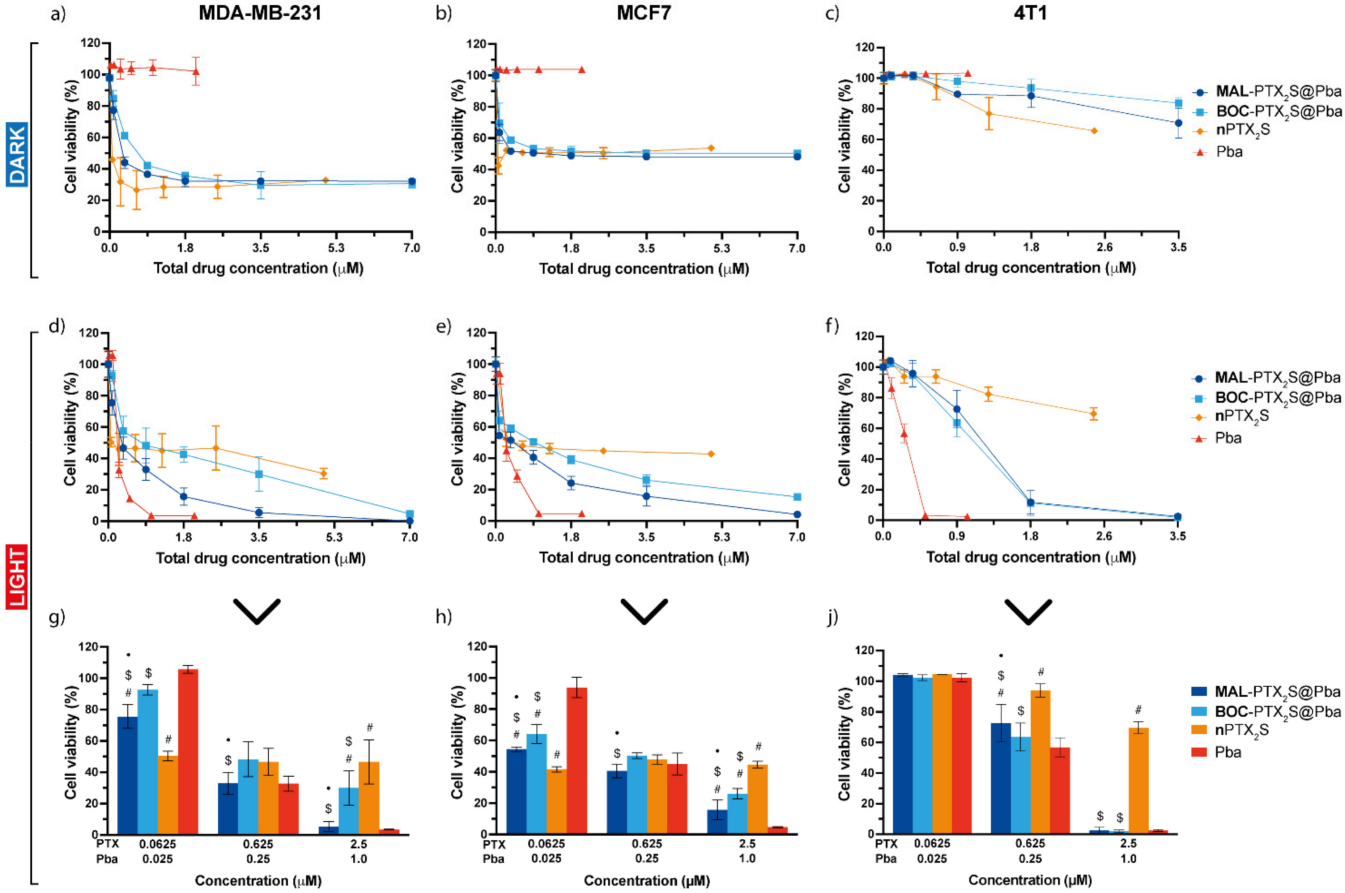
In vitro mono and combination therapy on cells cultured as monolayers. MDA-MB-231 (**a**,**d**,**g**), MCF7 (**b**,**e**,**h**) and 4T1 (**c**,**f**,**j**) cells were incubated for 24 h with the different drugs/drug combinations and maintained in the dark (**a**–**c**) or exposed to red light (1 J/cm^2^) (**d**–**f**). Cell viability was assessed with the MTS assay after additional 24 (MDA-MB-231, MCF7) or 48 h (4T1) of cells release in drug-free medium. To better compare the cytotoxic effects after light exposure, some concentrations have been extrapolated from the dose-response curves: (**g**) for MDA-MB-231, (**h**) for MCF7, and (**j**) for 4T1 cells. Total drug concentration is referred to PTX + Pba concentration. Data are expressed as mean percentage ± SD of at least three independent experiments, carried out in triplicate. Statistical significance was calculated applying the ANOVA Two-way with Bonferroni’s correction: ● significantly different from **BOC**-PTX_2_S@Pba; # significantly different from Pba; $ significantly different from **n**PTX_2_S.

Combination therapy results are reported in Figure 6, showing the dose–response curves of cells incubated for 24 h with drugs/nanoparticles, maintained in the dark (a–c) or irradiated with red light (total dose 1 J/cm^2^) (d–j), and assessed for viability 24 h (MDA-MB-231 and MCF7) or 48 h (4T1) after cell release in drug-free medium. IC_50_ values calculated for each treatment option are reported in Table 1.

Dose-response curves of cells not exposed to light (Figure 5a–c) showed the absence of Pba cytotoxicity in the dark in all cell lines, but confirmed a significant cell viability reduction, especially at very low PTX-equivalent doses, in MDA-MB-231 and MCF7 cells. Furthermore, a plateau is reached at PTX concentration near to the IC_50_ value in the abovementioned cell lines, while in 4T1 cells the IC_50_ was not even reached.

Remarkably, when PDT was part of the treatment (Figure 6d–h), a complete cell killing was achieved in all the three cell lines treated with Pba or Pba-loaded nanoparticles. In agreement with Pba uptake studies (Figure 5), the calculated IC_50_ values for human breast cancer (BC) cell lines (Table 1) were in the order Pba < **MAL**-PTX_2_S@Pba < **BOC**-PTX_2_S@Pba, referred to the total drugs dose. Importantly, from the analysis of the extrapolated cell viability data from the dose-response curves of MDA-MB-231 and MCF7 cells (Figure 6g,h), it can be observed that cell mortality determined by free Pba is comparable (or even lower when low drug doses are administered, e.g., 0.0625 and 0.025 μM PTX and Pba, respectively) to that elicited by **MAL**-PTX_2_S@Pba, even if the uptake of free Pba is significantly higher. These data can be ascribed to the contribution of PTX cytotoxicity, and are in agreement with a lower aggregation of Pba molecules once loaded into PTX prodrug-based nanoparticles (Figure S11) [30], thus resulting in an enhanced overall phototoxic effect. Of note, while in human BC cells the IC_50_ of **MAL**-PTX_2_S@Pba is about two times lower than that of **BOC**-PTX_2_S@Pba, their IC_50_ values are comparable in murine 4T1 cells (Table 1), very likely due to the scarce cytotoxic effect induced by PTX on this cell line. To exclude that the scarce cytotoxic response to nanoparticles was due to an inefficient prodrug’s activation in 4T1 cells, we assessed the cytotoxicity of free PTX formulated in standard conditions (e.g., DMSO), showing very limited efficacy, and comparable to that exerted by our nanosystems (Figure S12).

These data are in agreement with literature reports accounting for a limited efficacy of PTX on in vitro 4T1 cells [49,50] and on primary 4T1 tumor models [51].

**Figure 7:**
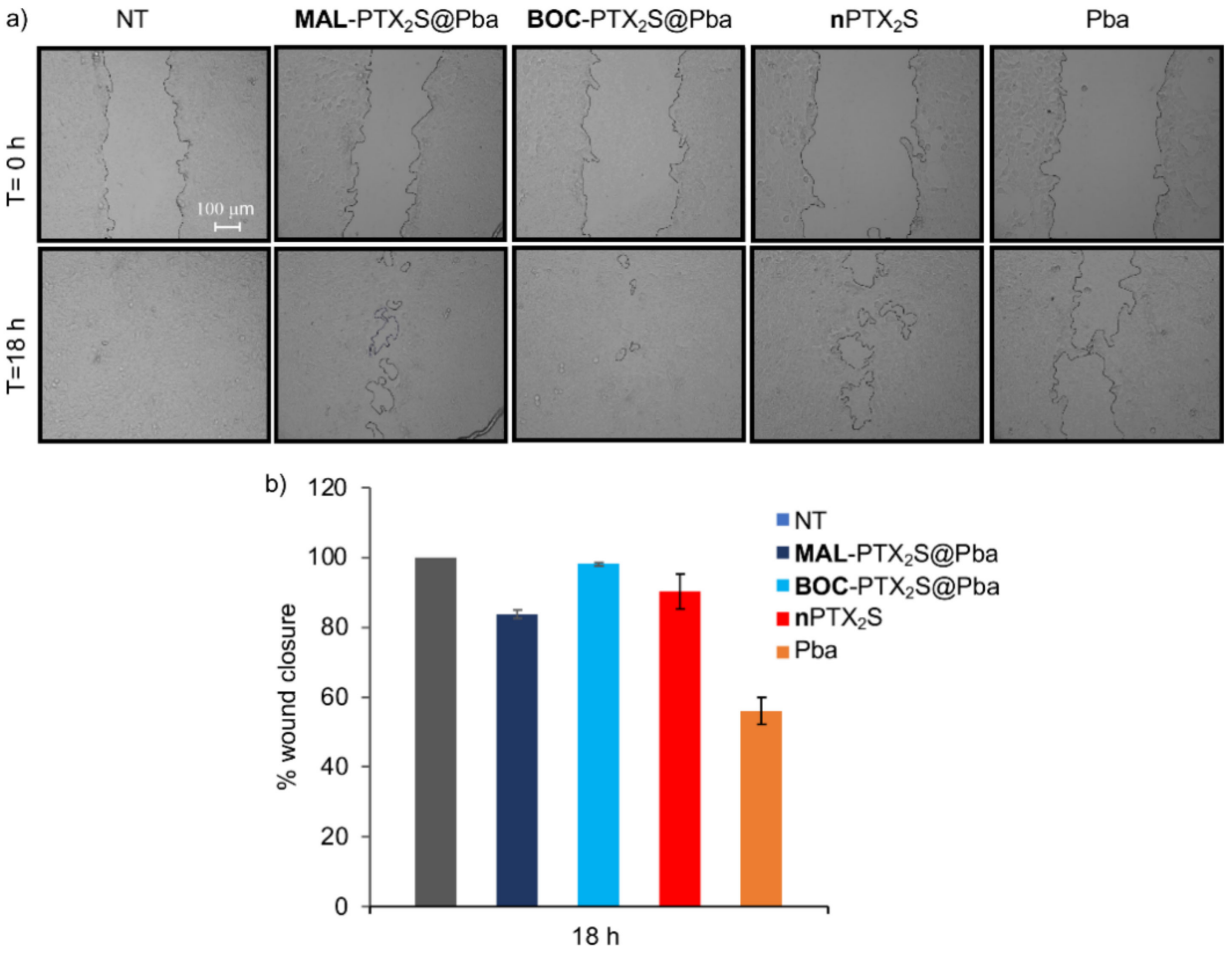
The motility of 4T1 cells was measured by wound healing assay. (**a**) Phase-contrast microscopy images (scale bar: 100 µm) at the beginning of the experiment (0 h) and at the end point (18 h). (**b**) Graph representing the percentage of wound closure.

A wound healing assay was also performed to investigate the ability **MAL**-PTX_2_S@Pba NPs to reduce 4T1 cells invasiveness. For this experiment and considering that murine breast cancer cells are highly aggressive, with intrinsic resistance to PTX and a fast-doubling time (<18 h), cells were treated with the different compounds (at the equimolar concentration of Pba 0.08 μM and 0.2 μM PTX), and a wound was generated after 4 h of dark incubation, followed by light irradiation (Figure 7).

The percentage of closure inhibition measured 18h post-PDT was 54% for free Pba while for **MAL**-PTX_2_S@Pba was 20%; of note, cells treatment with **BOC**-PTX_2_S@Pba and **n**PTX_2_S only slightly affected the closure, being the percentages quite comparable to untreated cells (Figure 7). Similar experiments performed on human breast cancer cells showed that, despite longer times were required for wound closure (30 h and 70 h for MD-MBA231 and MCF-7, respectively), **MAL**-PTX_2_S@Pba was the most effective in inhibiting the closure as compared with other treatments (Figure S13), overall suggesting that **MAL**-PTX_2_S@Pba NPs might have a potential to inhibit invasiveness and thus metastatic potential also in vivo.

To obtain more insight on the type of interaction between PTX and Pba once loaded into NPs in the three different cell lines, we performed a Compusyn analysis of the photo-toxicity data reported in Figure 6d–f, to calculate Combination Index (CI) using the median-effect principle, where CI < 1 indicates synergism; CI = 1 additivity, and CI > 1 antagonism [39]. As clearly visible from the results of the analysis reported in Figure S14, **MAL**-PTX_2_S@Pba determined a synergistic cell killing (CI < 1) for fraction affected (Fa) values higher than 0.5 in both MDA-MB-231 and MCF7 cells, whereas the effect of combo therapy using **BOC**-PTX_2_S@Pba was almost antagonistic. In agreement with the rather null cytotoxic effect determined by PTX in 4T1 cells, combination therapy determined CI > 1 in the entire interval of Fa values. Notwithstanding drugs interaction was not synergic in 4T1 cells, **MAL**-PTX_2_S@Pba NPs induced a higher PTX dose reduction index (DRI) value (Table 1) in 4T1 cells with respect to the human counterparts (e.g., 10.15, 0.53 and 2.23 for 4T1, MDA-MB-231 and MCF7 cells, respectively), whereas Pba DRI values obtained upon **MAL**-PTX_2_S@Pba treatment were more favorable for human cells as respect to 4T1 cells (e.g., Pba DRI of 3.92, 4.96 and 0.81 in MDA-MB-231, MCF7 and 4T1 cells, respectively).

### 3.6. In Vitro Combination Therapy with **MAL**-PTX_2_S@Pba in 3D-Models

The anticancer activity of **MAL**-PTX_2_S@Pba was also tested in multicellular spheroids derived from the triple negative BC cell lines 4T1 and MDA-MB-231. Despite the lack of vascularization of this in vitro model, it mimics the tumor architecture and environment more closely, thus providing important insights.

**Figure 8:**
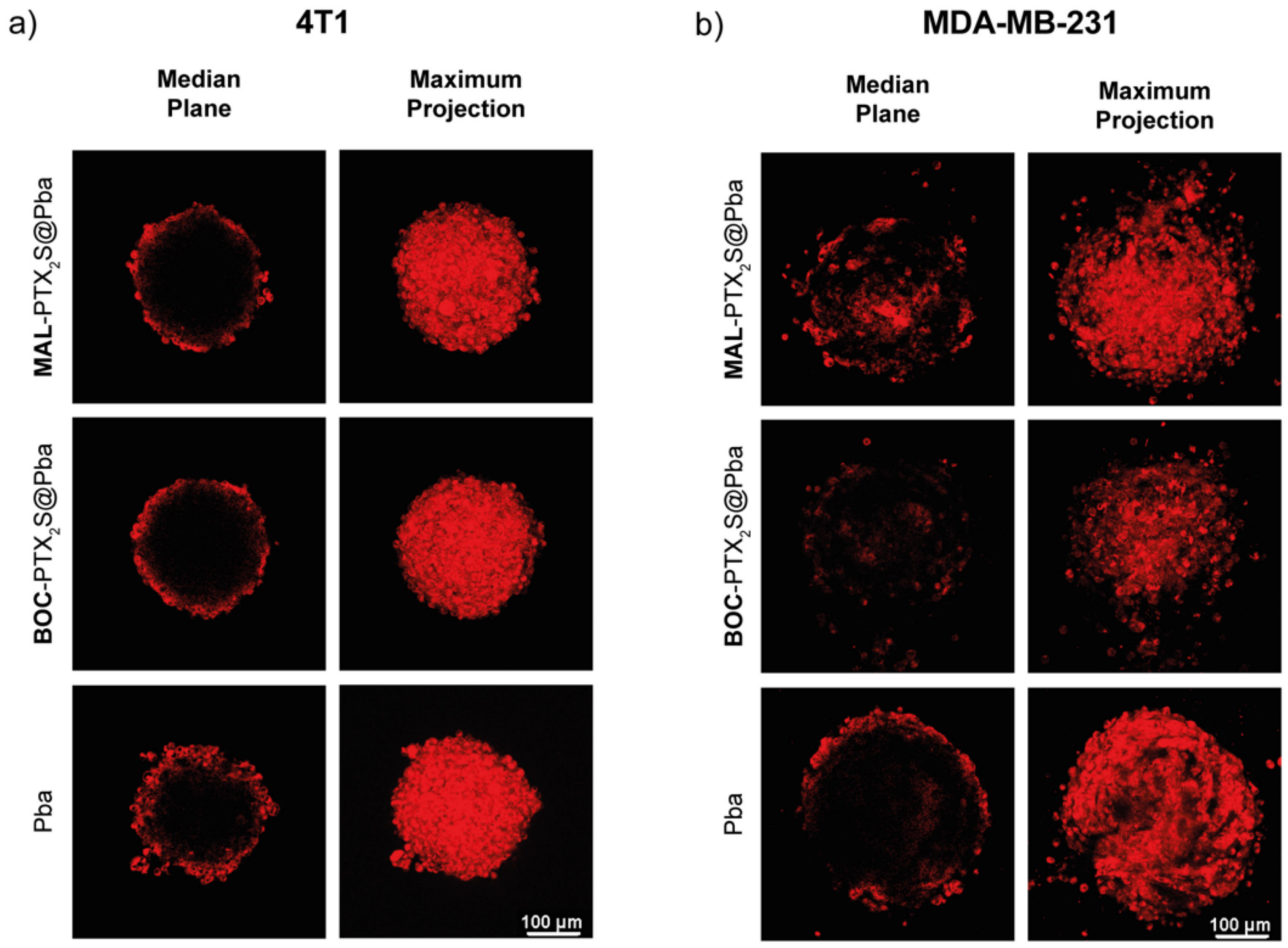
In vitro uptake of the different Pba formulations in 4T1 (**a**) and MDA-MB-231 (**b**) cells cultured as spheroids. Spheroids were incubated for 24 h with 0.5 μM of Pba dissolved in the standard solvent or loaded in **MAL**-PTX_2_S@Pba or in **BOC**-PTX_2_S@Pba and analyzed by confocal microscopy at the end of incubation time. Median plane images show Pba fluorescence distribution at the equatorial plane of each spheroid, whereas maximum projection images represent the fluorescence signal of the superimposition of about 50 different acquired focal planes. Scale bars: 100 μm.

To this end, the drug penetration in spheroids exposed to **MAL**-PTX_2_S@Pba, **BOC**-PTX_2_S@Pba and free Pba for 24 h was assessed by monitoring PS fluorescence by confocal microscopy (Figure 8). As visible especially in the maximum projection images of Figure 8a, Pba internalization extent in 4T1 spheroids was significantly higher for the free PS with respect to **MAL**-PTX_2_S@Pba and **BOC**-PTX_2_S@Pba, which similarly accumulated within the spheroid structure. However, as it can be observed from the median plane images, despite the different fluorescence intensity, the extent of PS penetration was similar for all Pba formulations tested, being confined in the outer rim of cells. The behavior is not surprising, being already observed for other photosensitizers alone or delivered with different systems [52,53], highlighting how the compactness of the spheroid structure and the presence of the extracellular matrix components could hamper drug diffusion.

Conversely, a remarkably different trend was observed for Pba penetration patterns in MDA-MB-231 spheroids treated with **MAL**-PTX_2_S@Pba and **BOC**-PTX_2_S@Pba, where Pba fluorescence was also detected in the inner parts of the spheroid’s structure, very likely because of the higher responsiveness of these cell lines to PTX, that in turn favored PS penetration. In agreement with this hypothesis, free Pba accumulated exclusively in the outer rim of cells of MDA-MB-231 spheroids, similarly to the tendency observed in 4T1 spheroids.

**Figure 9:**
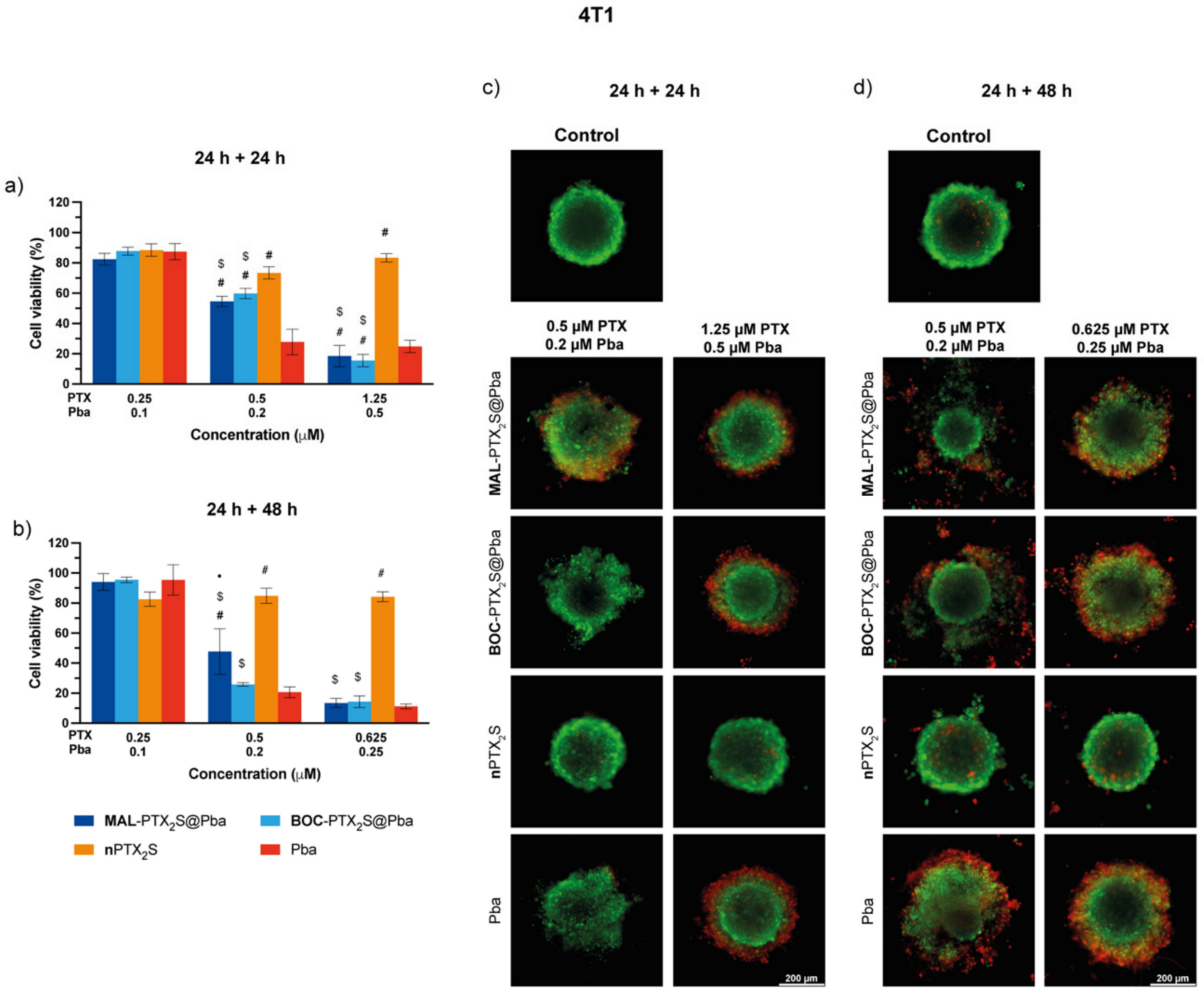
In vitro mono and combination therapy in 4T1 spheroids. The spheroids were incubated with the different drugs/combinations for 24 h and then irradiated with red light (1 J/cm^2^); cell viability was measured by means of ATP reduction (3D Glo assay) 24 (**a**) or 48 h (**b**) post-irradiation. Data are expressed as mean percentage ± SD of at least two independent experiments, carried out in quadruplicate. Statistical significance was calculated applying the ANOVA Two-way with Bonferroni’s correction: ● significantly different from **BOC**-PTX_2_S@Pba; # significantly different from Pba; $ significantly different from nPTX_2_S. Selected images of spheroids stained with the LIVE/DEAD kit and acquired 24 (**c**) or 48 h (**d**) after irradiation. Scale bars: 200 μm.

**Figure 10:**
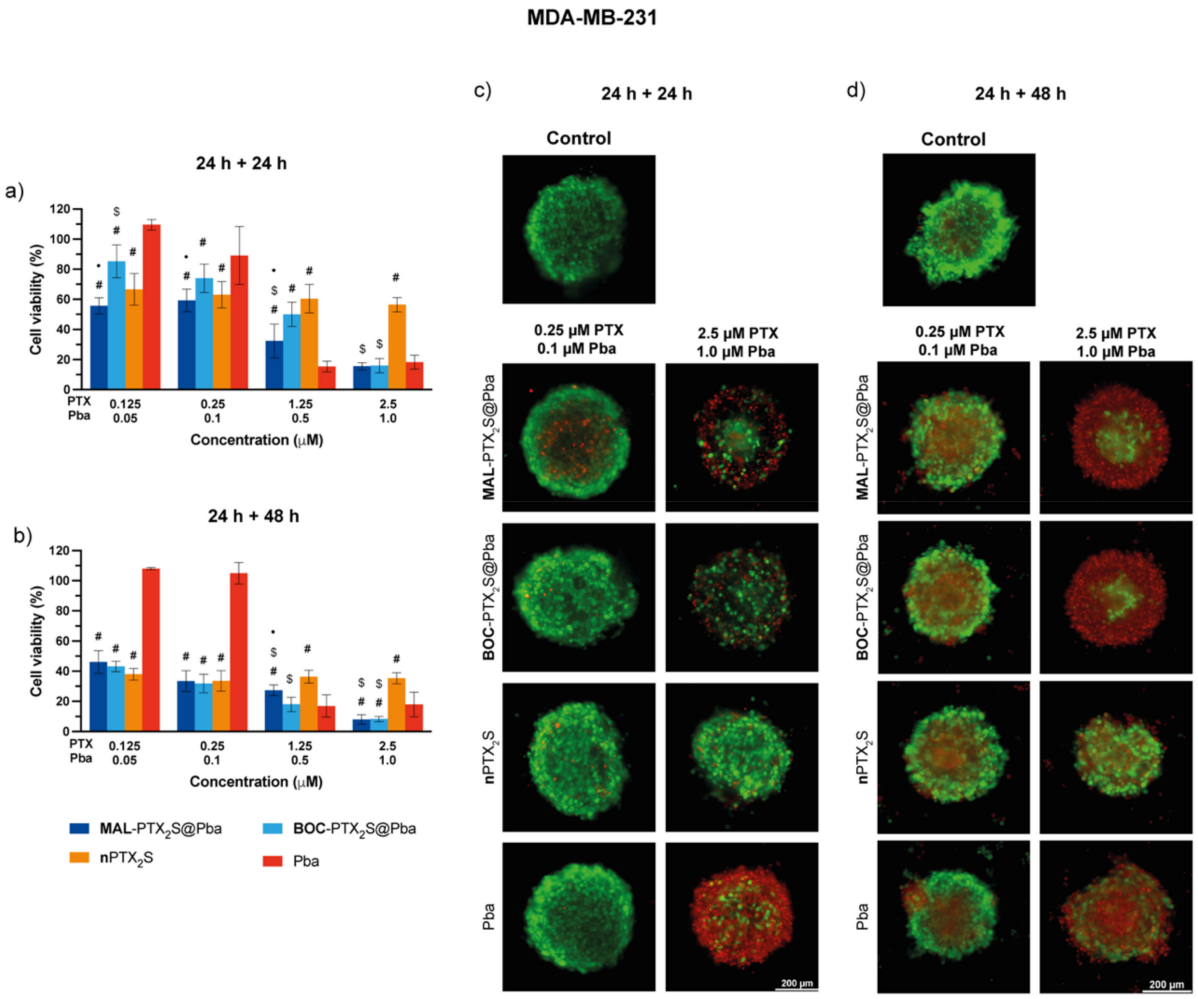
In vitro mono and combination therapy in MDA-MB-231 spheroids. The spheroids were incubated with the different drugs/combinations for 24 h and then irradiated with red light (1 J/cm^2^); cell viability was measured by means of ATP reduction (3D Glo assay) 24 (**a**) or 48 h (**b**) post-irradiation. Data are expressed as mean percentage ± SD of at least two independent experiments, carried out in quadruplicate. Statistical significance was calculated applying the ANOVA Two-way with Bonferroni’s correction: ● significantly different from **BOC**-PTX_2_S@Pba; # significantly different from Pba; $ significantly different from **n**PTX_2_S. Selected images of spheroids stained with the LIVE/DEAD kit and acquired with a fluorescence microscope 24 (**c**) or 48 h (**d**) after irradiation. Scale bars: 200 μm.

Combination therapy in the absence (Figures S15 and S16) and in the presence of light (Figure 9 and Figure 10) was performed in the three-dimensional in vitro models maintaining unaltered the incubation and irradiation protocol used with cell monolayers, and by assessing cell viability reduction through ATP level measurement (e.g., 3D Glo Assay) 24 or 48 h after irradiation. As expected, and in agreement with the results obtained in cells monolayers, 4T1 spheroids showed extreme resistance toward PTX formulations treatment [49,50,51] at both time points (Figure 9), whereas Pba activation with red light showed a progressive and dose-dependent loss of viability. As visible from graphs of Figure 9a,b and Table S4, reporting the IC_50_ values of the different formulations, **MAL**-PTX_2_S@Pba treatment did not significantly improve cell killing efficiency with respect to **BOC**-PTX_2_S@Pba, whereas free Pba showed the lowest IC_50_ value, in agreement to its higher uptake (Figure 8a).

To further deepen the correlation between the sites of drug accumulation and those of the cellular damages elicited by the treatments, live/dead microscopy analysis was carried out (Figure 9c,d and Figure S17 for the quantification of fluorescence intensities). The results clearly reveal the presence of dead cells (red fluorescence) mostly in the outer spheroid’s rims, accordingly to PS distribution, at both time points considered. Interestingly, the observation of images acquired at the 24 + 48 h time point, revealed an improved capacity of **MAL**-PTX_2_S@Pba treatment to promote spheroid disaggregation with respect to **BOC**-PTX_2_S@Pba and with respect to free Pba, although the overall extent of cell death was comparable. Moreover, as visible also from the results of Figure S17, the percentages of dead cells increased by increasing the observation time (e.g., 24 + 48 h time point), as for the trend observed for viability reduction (Figure 9a,b). These data suggest that combo therapy mediated by our nanoformulation could improve therapy outcome very likely by disrupting the interaction between cells and the extracellular matrix components, thus affecting tumor mass compactness.

Combination therapy in MDA-MB-231 spheroids (Figure 10) showed a 3-fold higher killing efficiency of **MAL**-PTX_2_S@Pba as respect to **BOC**-PTX_2_S@Pba (IC_50_ of 0.39 and 1.17 μM, respectively, Table S4) when cell viability was measured 24 h after light irradiation. Conversely, when cells viability was assayed 48 h post-irradiation, a significantly lower, but comparable, IC_50_ values were achieved. It is worth noticing that, at the 24 + 48 h time point, NPs-mediated treatment was more effective in reducing cell viability than single drugs (Table S4), confirming the effectiveness of our combined approach in this 3D tumor model. In agreement with the localization study (Figure 8), the live/dead analysis (Figure 10c,d and Figure S17c,d) revealed a clear concentration- and time-dependent increase in cells death not only in the spheroid periphery but also in the inner core, thus confirming that PTX cytotoxicity enhances drug penetration in the inner parts of the spheroids.

### 3.7. In Vivo Experiments

#### 3.7.1. Tumor Biodistribution in 4T1 Tumor-Bearing Mice

**Figure 11:**
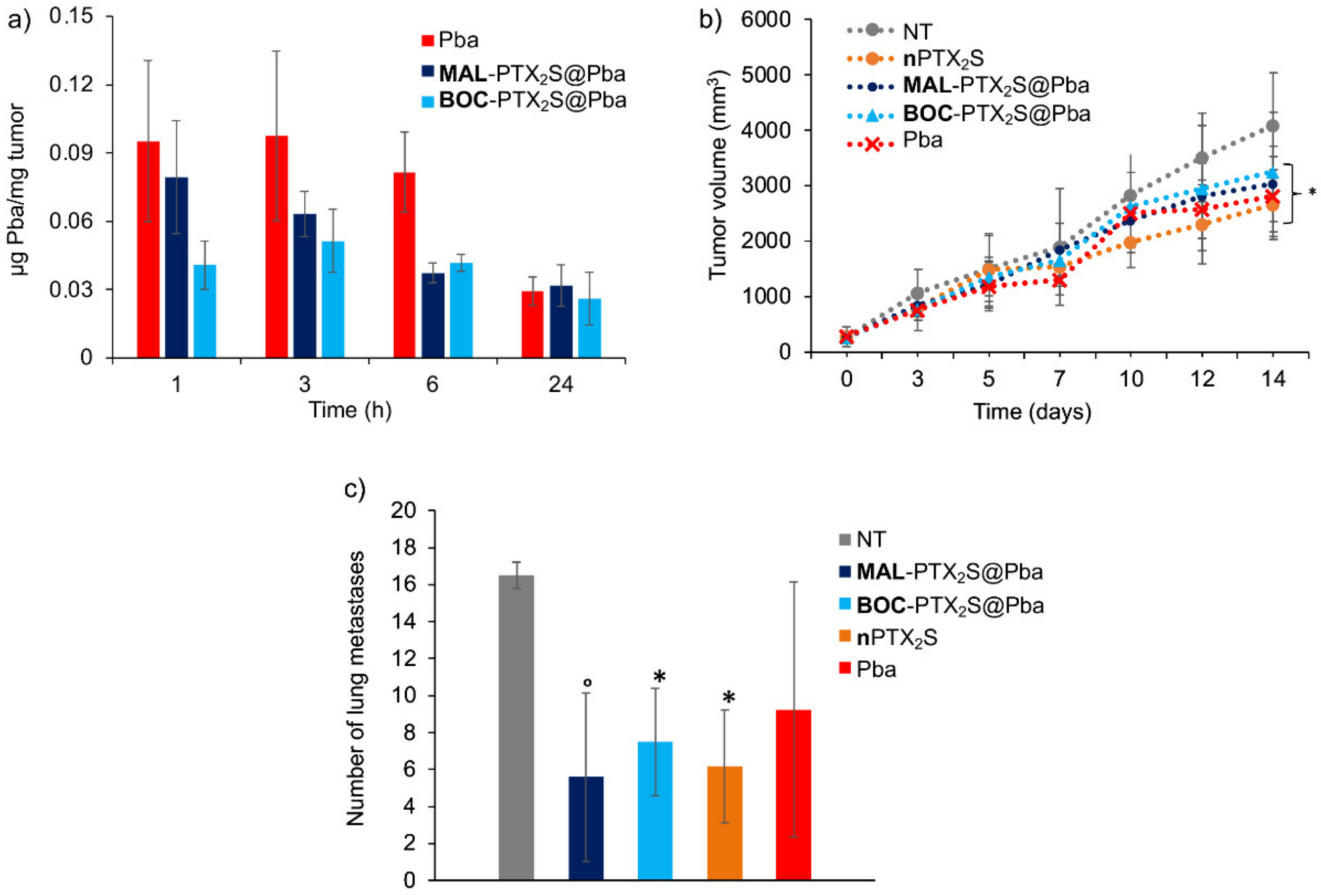
Effect of nanoparticles on 4T1 tumor-bearing mice. (**a**) Biodistribution of Pba in tumor after i.v. injection of Pba, **MAL**-PTX_2_S@Pba and **BOC**-PTX_2_S@Pba (containing 2.34 mg/Kg Pba); see Section 2.10.1 for experimental details. Each bar is the average of three measurements (three mice). (**b**) Antitumor efficacy of nanoparticles on primary tumor growth. Treated mice were injected on day 0 with a single i.v. treatment of 100 μL (injectable water) **MAL**-PTX_2_S@Pba, **BOC**-PTX_2_S@Pba, **n**PTX_2_S and Pba (2.34 mg/Kg Pba and 8.16 mg/Kg PTX). Three hours after injection, the tumor mass was irradiated with a 660 ± 5 nm laser (193 J/cm^2^). (* *p* < 0.05 vs. untreated group, ANOVA test). (**c**) Antitumor efficacy on the number of lung metastases. Lungs were harvested on the sacrifice day to examine the number of metastases by stereomicroscope. Results were expressed as mean ± SE (standard error). Symbols indicate the significant difference from the control group (° *p* < 0.01 and * *p* < 0.05, respectively, ANOVA Dunnett multiple comparison test).

To establish the best time for performing the PDT-treatment, the time-dependent tumor accumulation of Pba, **MAL**-PTX_2_S@Pba, and **BOC**-PTX_2_S@Pba was investigated in 4T1 tumor-bearing female BALB/c mice [40]. The amount of Pba recovered ex-vivo from tumors was measured as described in Section 2.9.1 and expressed in μg/mg tissue. As shown in Figure 11a, the highest Pba tumor accumulation was achieved 3 h after treatment for **MAL**-PTX_2_S@Pba, **BOC**-PTX_2_S@Pba and free Pba; therefore, this was selected as the time point for light irradiation in the following in vivo experiments.

#### 3.7.2. In Vivo Antitumor Efficacy

Considering our preliminary in vitro results, accounting for a higher PTX DRI in 4T1 cells treated with **MAL**-PTX_2_S@Pba (Table 1), as well as a reduced invasiveness potential mediated by our particles (Figure 7), we sought to preliminary assess in vivo whether a low PTX-PDT regimen could determine the reduction in the primary tumor and a decrease in pulmonary metastases formation.

The antitumor efficacy of **MAL**-PTX_2_S@Pba as respect to **BOC**-PTX_2_S@Pba, **n**PTX_2_S and free Pba was evaluated on 4T1 tumor-bearing female BALB/c mice model. When the tumor reached a volume of about 200 mm^3^ (6–8 mm, largest diameter), the mice were treated intravenously with the different compounds/formulation at a concentration of 2.34 mg/Kg and 8.16 mg/Kg for Pba and PTX, respectively. Three hours after administration, mice were irradiated with a 660 nm laser at 193 J/cm^2^. After the treatment, tumor growth was measured every 2 days by caliper.

The tumor growth curves from the day of treatment (day 0) to the day of sacrifice (day 14) (Figure 11b) show that all treated groups were significantly different from the untreated group (NT) (**MAL**-PTX_2_S@Pba, **BOC**-PTX_2_S@Pba and **n**PTX_2_S *p* < 0.01; Pba *p* = 0.05 by the two-way ANOVA test), whereas no significant difference was observed among the treated groups (Tukey’s test).

Moreover, as shown in Figure 11c, mice treated with **MAL**-PTX_2_S@Pba, **BOC**-PTX_2_S@Pba and **n**PTX_2_S, developed a lower number of lung metastases and were significantly different from the control group (*p* < 0.01, *p* < 0.05 and *p* < 0.05, respectively, ANOVA Dunnett multiple comparison test). However, no significant difference was observed between the **MAL**-PTX_2_S@Pba group and the other treatments.

As for the weight of metastases, a statistical difference was observed through the unpaired t-test between **MAL**-PTX_2_S@Pba (*p* = 0.0038), **BOC**-PTX_2_S@Pba (*p* = 0.006) and **n**PTX_2_S (*p* = 0.003) as respect to untreated group, as well as between **MAL**-PTX_2_S@Pba and **BOC**-PTX_2_S@Pba treatments (*p* = 0.007) (Figure S15). Overall, these very preliminary data show that treatment with **MAL**-PTX_2_S@Pba tends to reduce lung metastases (number and weight), thus posing encouraging bases for future in vivo validation with higher/repeated treatment doses. Indeed, it is worth noticing that notwithstanding our experimental setup included an extremely low drugs dose, i.e., 8.16 mg/Kg PTX and 2.34 mg/Kg Pba, with respect to literature data ranging between 20 to 50 mg/Kg [54], still our new **MAL**-PTX_2_S@Pba NPs significantly reduced mammary tumor growth while displaying encouraging signal in curbing lungs metastasis growth.

## 4. Conclusions

This study describes the synthesis, characterization, in vitro and preliminary in vivo assessment of novel nanoparticles, i.e., **MAL**-PTX_2_S@Pba, capable of in situ binding to circulating human serum albumin (HSA), and to exert combined photo- and chemo-toxicity on breast cancer cells. Particles are composed of pheophorbide a (Pba), and two paclitaxel (PTX) prodrugs endowed with different responsiveness to the elevated glutathione and reactive oxygen species levels typical of the tumor environment. It was successfully demonstrated that **MAL**-PTX prodrug efficiently binds HSA with a 94% yield, whereas the corresponding non-HSA binding counterpart, e.g., **BOC**-PTX, reached only a 4.6% reaction yield. Overall, the presented data confirm the efficacy of our approach both in 2D and 3D models of human breast cancer cells. Conversely, 4T1 murine cancer cells displayed high resistance to PTX treatment in vitro. Importantly, our preliminary low-dose in vivo treatment (8.16 mg/Kg PTX and 2.34 mg/Kg Pba; single treatment) showed a significant primary tumor reduction compared with controls, and most notably, to possibly reduce the formation of lung metastases. Altogether, these results set the basis for future preclinical investigations on using **MAL**-PTX_2_S@Pba nanoparticles to treat metastatic breast cancer.

## Figures and Tables

**Table 1:** IC_50_ and Dose-Reduction Index (DRI) values calculated by the Compusyn software from the photo-toxicity data (Figure 5d–f) of cells exposed to the different drug formulations/combinations. The DRI values were calculated for cells exposed to combination therapy and indicate how many folds the concentration of each single drug can be reduced to obtain a survival value (Fa, Fraction affected) of 50%.

Drug Formulation	IC_50_ (µM)			DRI					
	MDA-MB-231	MCF7	4T1	MDA-MB-231		MCF7		4T1	
				Pba	PTX	Pba	PTX	Pba	PTX
nPTX_2_S	0.11	0.41	-	-	-	-	-	-	-
Pba	0.22	0.26	0.26	-	-	-	-	-	-
**MAL**-PTX_2_S@Pba	0.31	0.23	1.09	3.92	0.53	4.96	2.23	0.81	10.15
**BOC**-PTX_2_S@Pba	0.83	0.55	1.01	1.47	0.19	2.47	1.11	0.86	10.86

## Data Availability

The data presented in this study are available in this article (and supplementary material).

## Supplementary Materials

The following are available online at https://www.mdpi.com/article/10.3390/cancers14040877/s1, Figure S1. ^1^H-NMR spectrum of derivative 4. Figure S2. ^1^H-NMR spectrum of **MAL** derivative. Figure S3. ^1^H-NMR spectrum of **BOC** derivative. Figure S4. Optimization of nanoparticles preparation. Figure S5. TEM image of **BOC**-PTX_2_S@Pba nanoparticle. Figure S6. Particle size stability of **BOC**-PTX_2_S@Pba NPs in FBS 20% in PBS pH 7.4 as determined by DLS analysis. Figure S7. HPLC-UV chromatograms at 228 nm for the release kinetics of **MAL**-PTX_2_S@Pba NPs. Figure S8. Kinetic evolution of relative peak areas for PTX_2_S and PTX as determined by HPLC-UV analysis on PTX_2_S solutions in different redox conditions. Figure S9. Dose and time-dependent adduct formation between **MAL** and HSA. Figure S10. In vitro uptake of the different Pba formulations in MCF10A cells cultured as monolayers. Figure S11. Absorption spectra of 10 μM Pba, **MAL**-PTX_2_S@Pba and **BOC**-PTX_2_S@Pba diluted in water. Figure S12. Cytotoxicity measured in 4T1 cells incubated with increasing concentrations of free PTX. Figure S13. Motility of MCF-7 and MDA-MB-231 cells measured by wound healing assay. Figure S14. Combination index analysis. Figure S15. Dark cytotoxicity measured in 4T1 spheroids incubated with the different drugs/combinations for 24 h. Figure S16. Dark cytotoxicity measured in MDA-MB-231 spheroids incubated with the different drugs/combinations for 24 h. Figure S17. Quantification of fluorescence intensity in 4T1 and MDA-MB-231 spheroids stained with the LIVE/DEAD kit. Table S1. List of PTX derivatives observed in HPLC-UV analysis. Table S2. List of Pba derivatives observed in HPLC-UV analysis. Table S3. Cumulative peak areas and released amounts for PTX and Pba at different dialysis times, as determined by HPLC-UV analysis in release experiments on **MAL**-PTX_2_S@Pba NPs. Table S4. IC50 values calculated by the Compusyn software from the photo-toxicity data of MDA-MB-231 and 4T1 spheroids exposed to the different drug formulations/combinations.
