# Supplementary material for: HSA-Binding Prodrugs-Based Nanoparticles Endowed with Chemo and Photo-Toxicity against Breast Cancer
Source: Cancers (Basel). 2022 Feb 10;14(4):877. doi: 10.3390/cancers14040877 (PMC8870514; doi:10.3390/cancers14040877)
Supplement: Supplementary file 1 [file cancers-14-00877-s001.zip › cancers-1537208-supplementary.pdf]

HSA-binding prodrugs-based nanoparticles endowed with chemo and photo-toxicity against breast cancer

Valentina Rapozzi[1,†], Francesca Moret[2,†], Luca Menilli[2], Andrea Guerrini[3], , Daniele Tedesco[3], Marina Naldi[4], Manuela Bartolini[4], Mariachiara Gani[1], Sonia Zorzet[5], Marta Columbaro[6], Celeste Milani[2], Cecilia Martini[3], Claudia Ferroni[3,*] and Greta Varchi [3,*]

Supplementary Information

Table of Content

Supplementary Figures

Figure S1. ^{1}H-NMR spectrum of derivative **4** (500 MHz, $CDCl_3$). 3

Figure S2. ^{1}H-NMR spectrum of **MAL** derivative (500 MHz, DMSO). 3

Figure S3. ^{1}H-NMR spectrum of **BOC** derivative (500 MHz, DMSO). 4

Figure S4. Optimization of nanoparticles preparation. a) Size trend by varying the % amount of PTX_2S and **MAL**; b) Polydispersity index (PDI) trend by varying the % amount of PTX_2S and **MAL**. 4

Figure S5. TEM image of **BOC**-PTX_2S@Pba nanoparticle (scale bar 1mm). 5

Figure S6. Particle size stability of **BOC**-PTX_2S@Pba NPs in FBS 20% in PBS pH 7.4, v/v (green dotted line), and HSA 35 mg/mL in PBS pH 7.4, v/v (orange dotted line), as determined by DLS analysis. 5

Figure S7. HPLC-UV chromatograms at 228 nm for the release kinetics of **MAL**-PTX_2S@Pba NPs, as obtained by dialysis experiments in redox-neutral (medium **A**), strongly reducing (medium **C**) and strongly oxidizing (medium **E**) conditions. Experimental conditions are reported in the Main Text (Paragraph 2.5). 6

Figure S8. Kinetic evolution of relative peak areas for PTX_2S (*left*) and PTX (*right*), as determined by HPLC-UV analysis on PTX_2S solutions in different redox conditions. *Diamonds*: redox-neutral; *empty circles*: mildly reducing; *filled circles*: strongly reducing; *empty squares*: mildly oxidizing; *filled squares*: strongly oxidizing. 7

Figure S9. Dose and time-dependent adduct formation between **MAL** and HSA. 8

Figure S10. In vitro uptake of the different Pba formulations in MCF10A cells cultured as monolayers. Cells were incubated for 1 or 4h with 0.25 µM of Pba dissolved in the standard solvent or loaded in **MAL**-PTX_2S@Pba or in **BOC**-PTX_2S@Pba, and the uptake of Pba was measured by flow cytometry at the end of incubation times. Data are expressed as mean percentage ± SD of least two independent experiments, carried out in triplicate. Statistical significance was calculated applying the two-way ANOVA with Bonferroni's correction: # significantly different from Pba. 8

Figure S11. Absorption spectra of 10 µM Pba, **MAL-**PTX_2S@Pba and **BOC**-PTX_2S@Pba diluted in water. 9

Figure S12. Cytotoxicity measured in 4T1 cells incubated with increasing concentrations of free PTX for 24h and release in drug-free medium for additional 48h before assessing cell viability with the MTS assay. Data are expressed as mean percentage ± SD of least three independent experiments, carried out in triplicate. 9

Figure S13. The motility of MCF-7 cells (a) and MDA-MB-231 cells (b) was measured by wound healing assay. Phase-contrast microscopy images (scale bar: 100 µM) at the beginning of the experiment (0h) and the end point (70h for MCF-7 and 30h for MDA-MB-231 cells). 9

Figure S14. Combination index analysis. Fa-CI plots of Combination Index vs. Fa relative to MDA-MB-231, MCF-7 and 4T1 cells treated with the combination of PTX and Pba loaded in **MAL**-PTX_2S@Pba or **BOC**-PTX_2S@Pba. Data reported in Fig. 5 d-f of the main text were analyzed with the Compusyn software and Fa-CI plots derived. 10

Figure S15. Dark cytotoxicity measured in 4T1 spheroids incubated with the different drugs/combinations for 24h and maintaned in drug-free medium for additional 24 (a) or 48 (b) h before assessing cell viability by measuring ATP levels with the 3D Glo assay. Data are expressed as mean percentage ± SD of at least two independent experiments, carried out in quadruplicate. Statistical significance was calculated applying the ANOVA Two-way with Bonferroni's correction: # significantly different from Pba. 10

Figure S16. Dark cytotoxicity measured in MDA-MB-231 spheroids incubated with the different drugs/combinations for 24h and maintaned in drug-free medium for additional 24 (a) or 48 (b) h before assessing cell viability by measuring ATP levels with the 3D Glo assay. Data are expressed as mean percentage ± SD of at least two independent experiments, carried out in quadruplicate. Statistical significance was calculated applying the ANOVA Two-way with Bonferroni's correction: • significantly different from **BOC**-PTX_2S@Pba; # significantly different from Pba; $ significantly different from **n**PTX_2S. 10

Figure S17. Quantification of fluorescence intensity in 4T1 and MDA-MB-231 spheroids stained with the LIVE/DEAD kit. The images reported in Figure 9 (4T1 spheroids) and 10 (MDA-MB-231 spheroids) were quantified using ImageJ software in order to quantify the fluorescence intensity from live cells (stained by calcein AM, green fluorescence) and dead cells (stained by Ethidium homodimer-1 (EthD-1), red fluorescence). a) quantification of images in Fig. 9 c; b) quantification of images in Fig. 9 d; c) quantification of images in Fig. 10 c; d) quantification of images in Fig. 10 d. 11

Supplementary tables

Table S1. List of PTX derivatives observed in HPLC-UV analysis. 5

Table S2. List of Pba derivatives observed in HPLC-UV analysis. Experimental conditions are reported in the Main Text (Paragraph 2.5)...6

Table S3. Cumulative peak areas and released amounts for PTX and Pba at different dialysis times, as determined by HPLC-UV analysis in release experiments on **MAL**-PTX_2S@Pba NPs. Experimental conditions are reported in the Main Text (Paragraph 2.5). 7

Table S4. IC_{50} values calculated by the Compusyn software from the photo-toxicity data (Figure 6 g,j) of MDA-MB-231 and 4T1 spheroids exposed to the different drug formulations/combinations and irradiated with 1 J/cm^2 of red light. 12

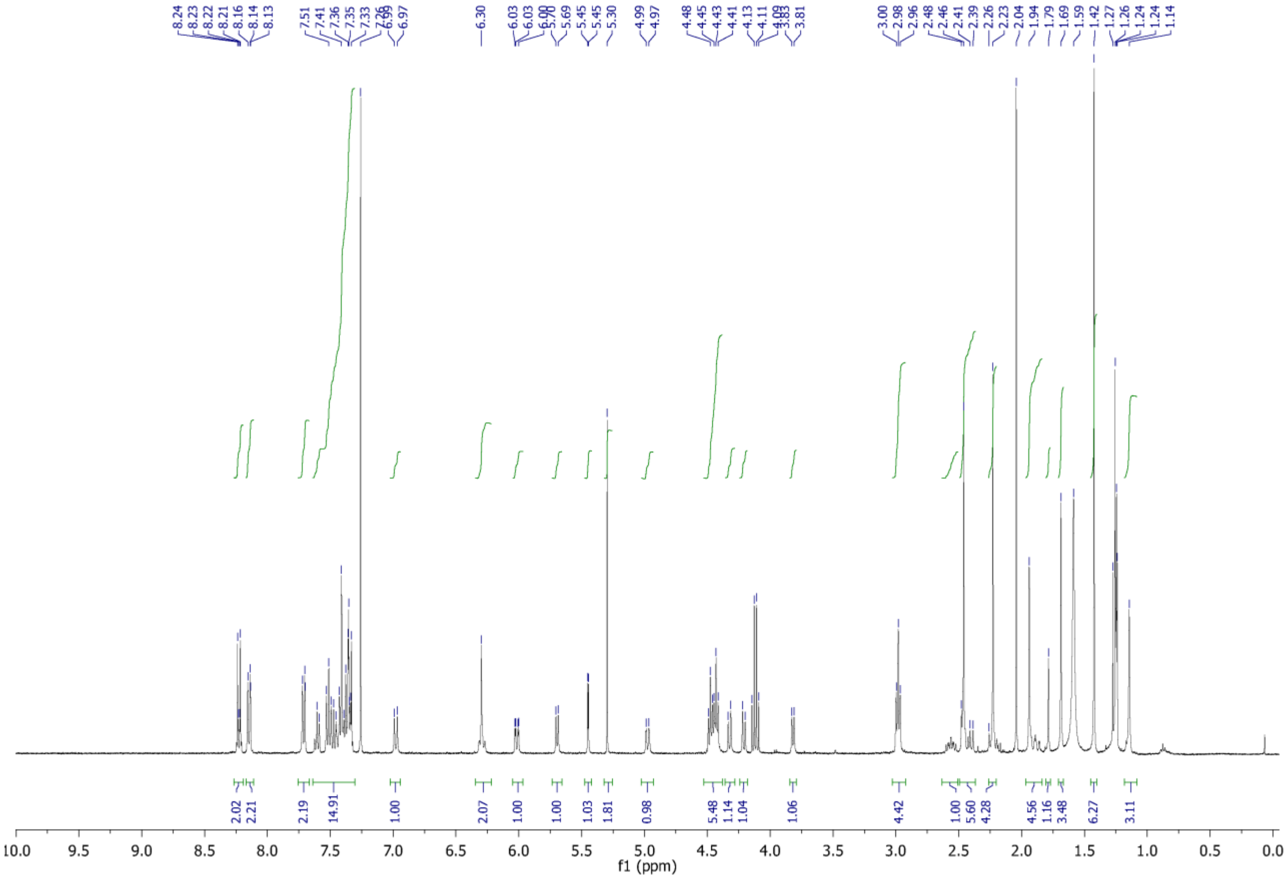

Figure S1. ^{1}H-NMR spectrum of derivative **4** (500 MHz, $CDCl_3$).

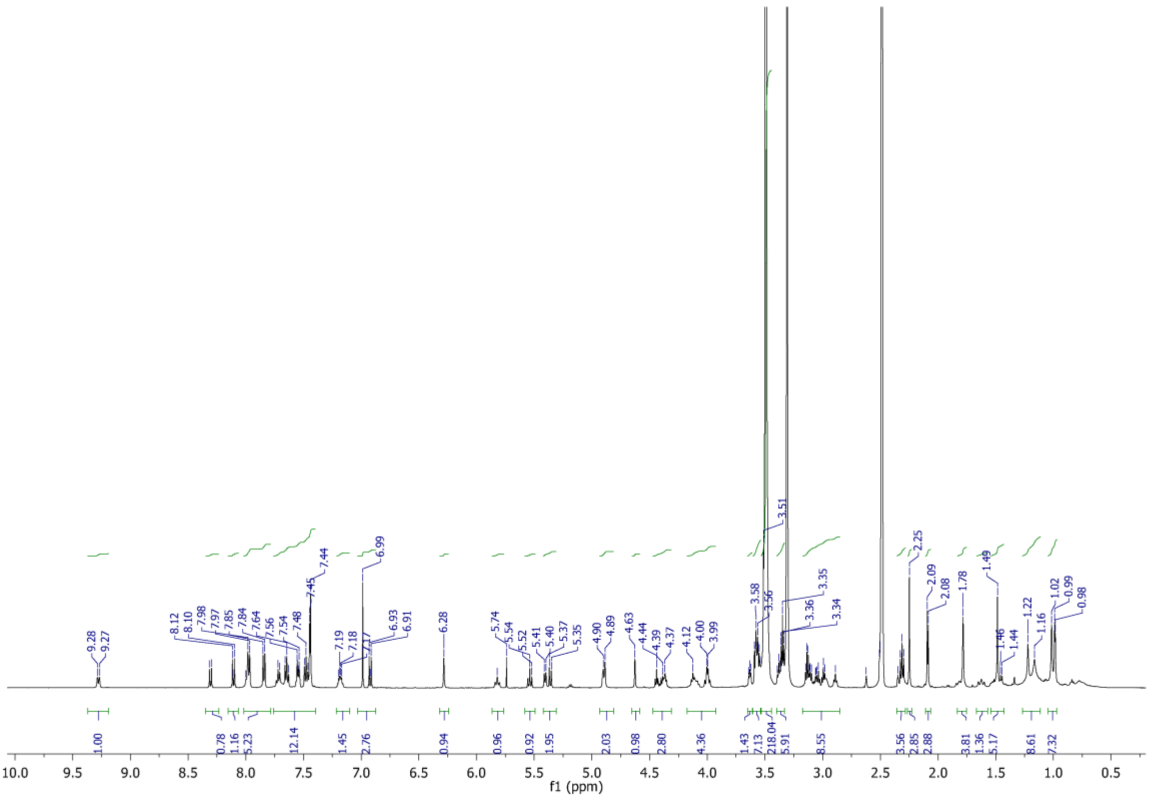

Figure S2. ^{1}H-NMR spectrum of **MAL** derivative (500 MHz, DMSO).

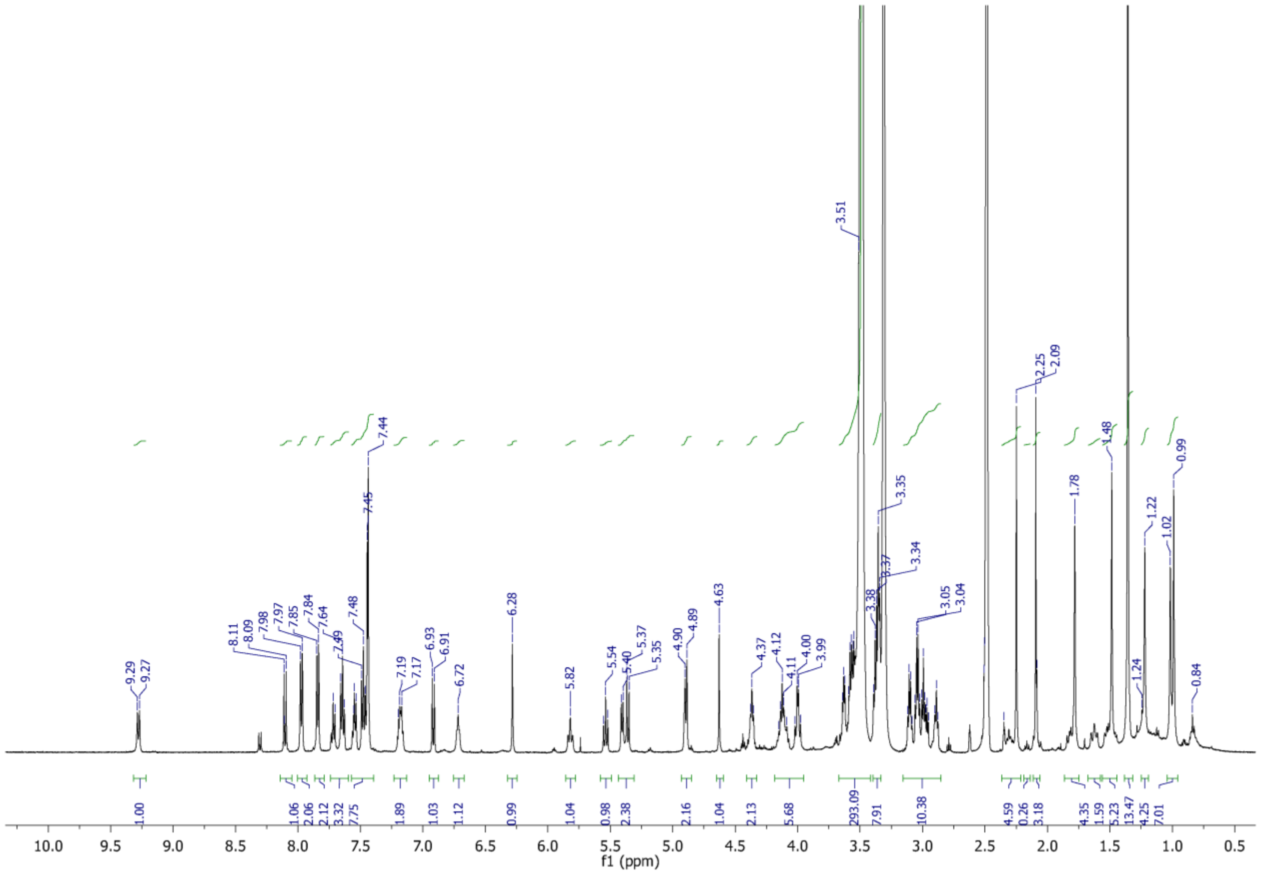

Figure S3. ^{1}H-NMR spectrum of **BOC** derivative (500 MHz, DMSO).

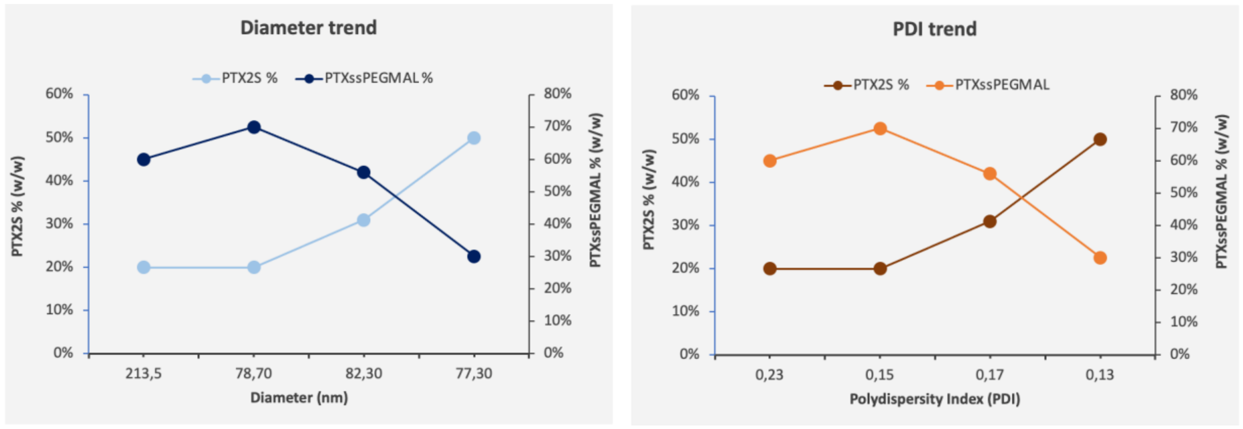

Figure S4. Optimization of nanoparticles preparation. a) Size trend by varying the % amount of PTX_2S and **MAL**; b) Polydispersity index (PDI) trend by varying the % amount of PTX_2S and **MAL**.

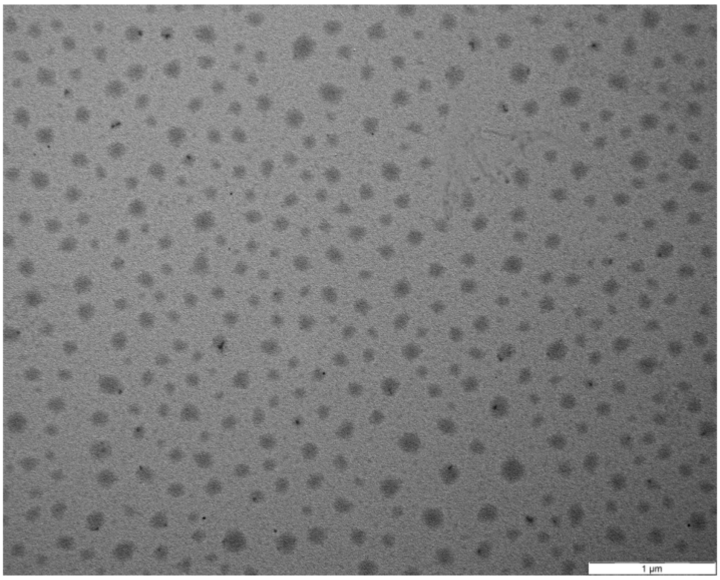

Figure S5. TEM image of **BOC**-PTX2S@Pba nanoparticle (scale bar 1mm).

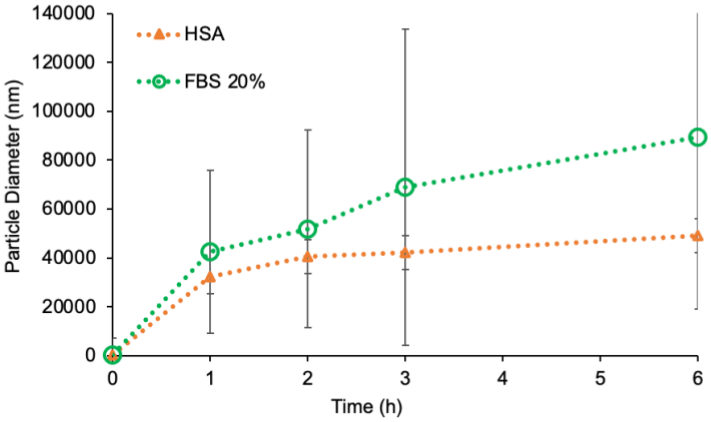

Figure S6. Particle size stability of **BOC**-PTX2S@Pba NPs in FBS 20% in PBS pH 7.4, v/v (*green dotted line*), and HSA 35 mg/mL in PBS pH 7.4, v/v (*orange dotted line*), as determined by DLS analysis.

Table S1. List of PTX derivatives observed in HPLC-UV analysis.
Experimental conditions are reported in the Main Text (Paragraph 2.5).

Used in cumulative peak area	t_R **(min)**	**Identity**	**Notes**
Paclitaxel dimer (**PTX2S**)	6.546	verified	
Paclitaxel (**PTX**)	4.744	verified	
7-Epipaclitaxel (**ePTX**)	5.257	verified	
Baccatin III (**B3**)	3.308	verified	
Paclitaxel dimer fragment 1	4.769	proposed	shoulder to the PTX peak, disappearing at long times
Paclitaxel dimer fragment 2	5.044	proposed	more intense in oxidizing conditions
Paclitaxel dimer fragment 3	5.725	proposed	less intense in oxidizing conditions
10-Deacetylpaclitaxel (**dPTX**)	4.135	proposed	more intense in oxidizing conditions
10-Deacetyl-7-epipaclitaxel (**dePTX**)	4.383	proposed	only in oxidizing conditions
Not used in cumulative peak area	t_R **(min)**	**Identity**	**Notes**
Paclitaxel side chain (**PSC**)	2.250	proposed	
unknown derivative	2.386	—	only in strongly oxidizing conditions
possible **MAL** derivative	2.516	—	very intense, only in strongly reducing conditions from **MAL**-PTX2S@Pba NPs
unknown derivative	2.586	—	only from **MAL**-PTX2S@Pba NPs

Table S2. List of Pba derivatives observed in HPLC-UV analysis. Experimental conditions are reported in the Main Text (Paragraph 2.5).

	t_R **(min)**	**HRMS (ESI,** ***m/z*****)** $[M\text{-}H]^-$	**Notes**
Pheophorbide a (**Pba**)	8.238	591.2618	
13^2-Epipheophorbide a (**ePba**)	8.526	591.2622	present in the commercial mixture of stereoisomers
unknown isomer	6.846	591.2621	trace amounts in the commercial mixture of stereoisomers
oxidized derivative	8.035	607.2571	
oxidized derivative	7.048	607.2575	
di-oxidized derivative	7.494	623.2518	
di-oxidized dehydrogenated derivative	7.358	621.2365	no absorption due to the porphyrin Q^{y}_{0-0} band (~650 nm)

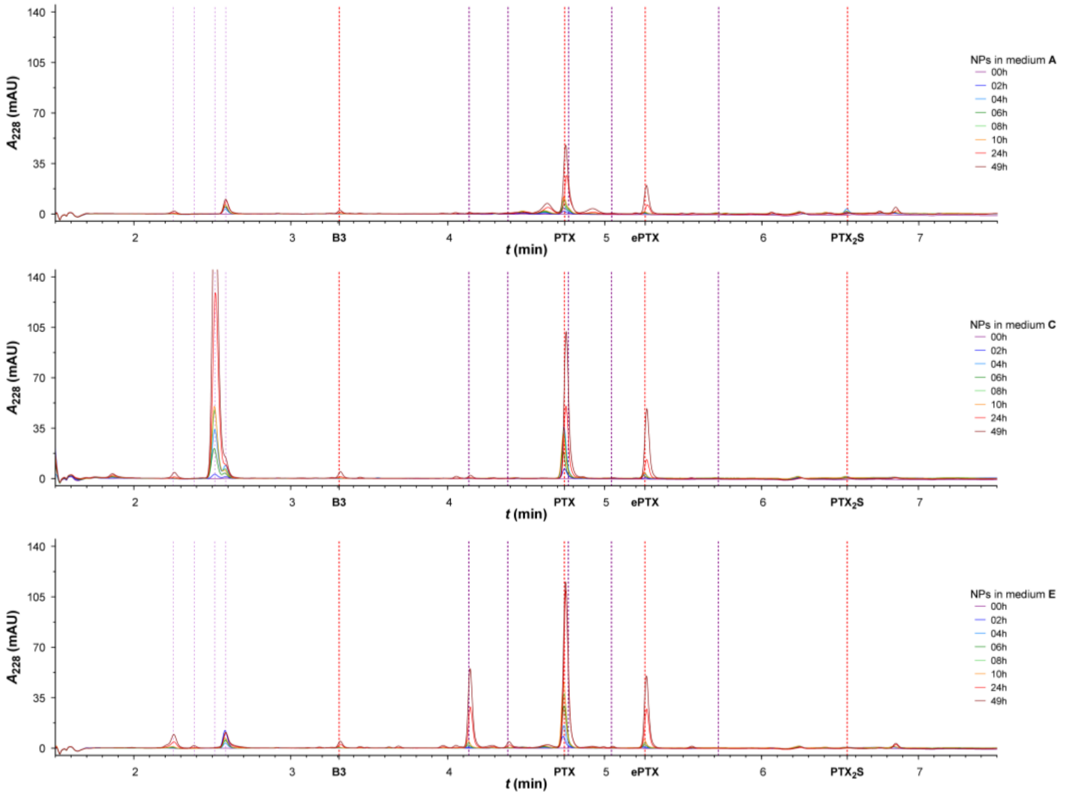

Figure S7. HPLC-UV chromatograms at 228 nm for the release kinetics of **MAL**-PTX_2S@Pba NPs, as obtained by dialysis experiments in redox-neutral (medium **A**), strongly reducing (medium **C**) and strongly oxidizing (medium **E**) conditions. Experimental conditions are reported in the Main Text (Paragraph 2.5).

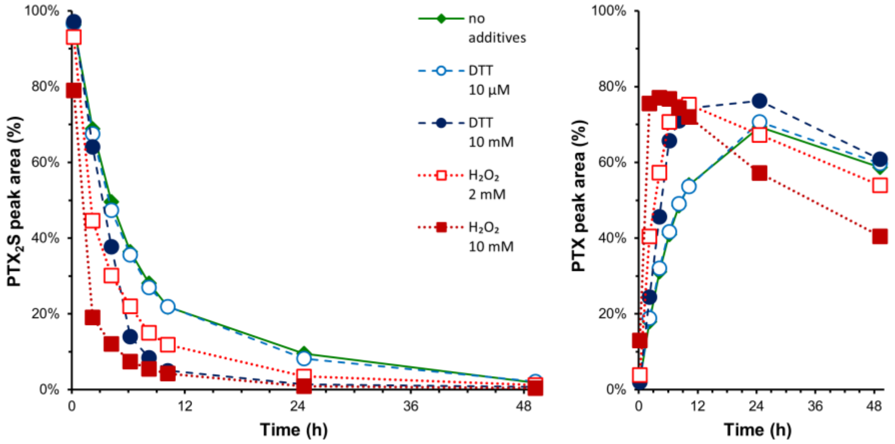

Figure S8. Kinetic evolution of relative peak areas for PTX_2S (*left*) and PTX (*right*), as determined by HPLC-UV analysis on PTX_2S solutions in different redox conditions. *Diamonds*: redox-neutral; *empty circles*: mildly reducing; *filled circles*: strongly reducing; *empty squares*: mildly oxidizing; *filled squares*: strongly oxidizing. Redox conditions are described in detail in the Main Text (Paragraph 2.5).

Table S3. Cumulative peak areas and released amounts for PTX and Pba at different dialysis times, as determined by HPLC-UV analysis in release experiments on **MAL**-PTX_2S@Pba NPs. Experimental conditions are reported in the Main Text (Paragraph 2.5).

Release medium **A**	**PTX** *		**Pba** **	
**Dialysis time (h)**	**Cumulative peak area**	**Release (% w/w)**	**Cumulative peak area**	**Released (% w/w)**
0.176	1959	0.2%	0	0.0%
2.170	6836	0.5%	33924	1.2%
4.178	18894	1.5%	105976	3.7%
6.179	23347	1.8%	114554	4.0%
8.181	31305	2.5%	137474	4.8%
10.182	36086	2.9%	158613	5.5%
24.631	81972	6.5%	206830	7.2%
49.179	162280	12.8%	314437	10.9%
Release medium **B**	**PTX** *		**Pba** **	
**Dialysis time (h)**	**Cumulative peak area**	**Released (% w/w)**	**Cumulative peak area**	**Released (% w/w)**
0.192	2314	0.2%	0	0.0%
2.185	9552	0.8%	34247	1.2%
4.195	18428	1.5%	96766	3.3%
6.197	25900	2.1%	120146	4.2%
8.198	34209	2.7%	145136	5.0%
10.199	45769	3.6%	170888	5.9%
24.647	102577	8.1%	280563	9.7%
49.196	165702	13.1%	358040	12.4%
Release medium **C**	**PTX** *		**Pba** **	
**Dialysis time (h)**	**Cumulative peak area**	**Released (% w/w)**	**Cumulative peak area**	**Released (% w/w)**
0.190	1528	0.1%	0	0.0%
2.184	18757	1.5%	28617	1.0%
4.195	82483	6.5%	57885	2.0%
6.197	48294	3.8%	71536	2.5%
8.198	85516	6.8%	76915	2.7%
10.200	77280	6.1%	90032	3.1%
24.648	144676	11.5%	124442	4.3%
49.197	346656	27.4%	239708	8.3%
Release medium **D**	**PTX** *		**Pba** **	
**Dialysis time (h)**	**Cumulative peak area**	**Release (% w/w)**	**Cumulative peak area**	**Release (% w/w)**
0.189	2161	0.2%	224	0.0%
2.183	5912	0.5%	19368	0.7%
4.195	19890	1.6%	68715	2.4%

6.197	34608	2.7%	115358	4.0%
8.198	47484	3.8%	140194	4.9%
10.200	59457	4.7%	150739	5.2%
24.648	135206	10.7%	219564	7.6%
49.198	217738	17.2%	249274	8.6%

Release medium **E**	**PTX ***		**Pba ****	
**Dialysis time (h)**	**Cumulative peak area**	**Released (% w/w)**	**Cumulative peak area**	**Released (% w/w)**
0.188	1269	0.1%	0	0.0%
2.182	20341	1.6%	39110	1.4%
4.195	38714	3.1%	65896	2.3%
6.197	72190	5.7%	98402	3.4%
8.198	96226	7.6%	116246	4.0%
10.200	121234	9.6%	133581	4.6%
24.648	356625	28.2%	239460	8.3%
49.199	491686	38.9%	316226	10.9%

* Cumulative peak area for PTX (30 µg/mL in release medium **A**): 1054191; maximum PTX concentration from total content of **MAL**-PTX$_2$S@Pba NP samples: 35.95 µg/mL.
** Cumulative peak area for Pba (15 µg/mL in release medium **A**): 4386975; maximum Pba concentration from total content of **MAL**-PTX$_2$S@Pba NP samples: 9.88 µg/mL.

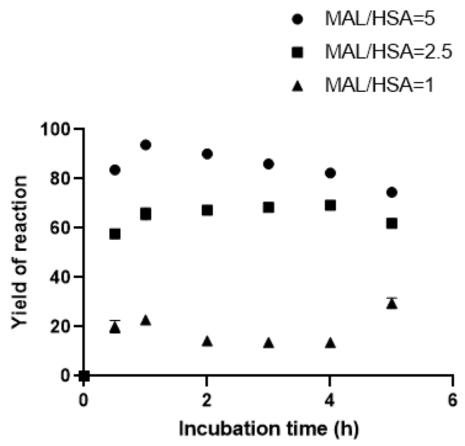

Figure S9. Dose and time-dependent adduct formation between **MAL** and HSA.

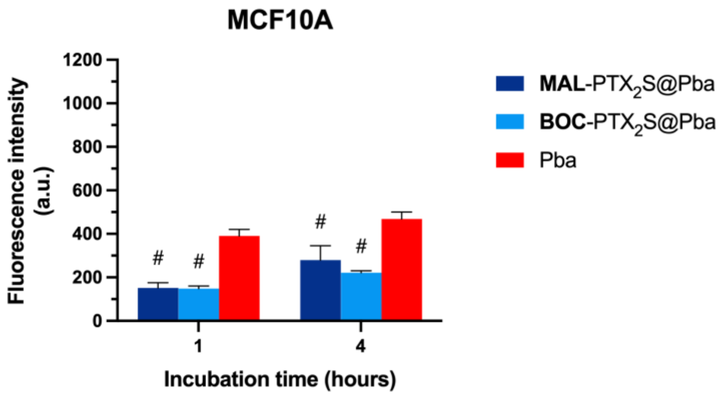

Figure S10. *In vitro* uptake of the different Pba formulations in MCF10A cells cultured as monolayers. Cells were incubated for 1 or 4h with 0.25 µM of Pba dissolved in the standard solvent or loaded in **MAL**-PTX$_2$S@Pba or in **BOC**-PTX$_2$S@Pba, and the uptake of Pba was measured by flow cytometry at the end of incubation times. Data are expressed as mean percentage ± SD of least two independent experiments, carried out in triplicate. Statistical significance was calculated applying the two-way ANOVA with Bonferroni's correction: # significantly different from Pba.

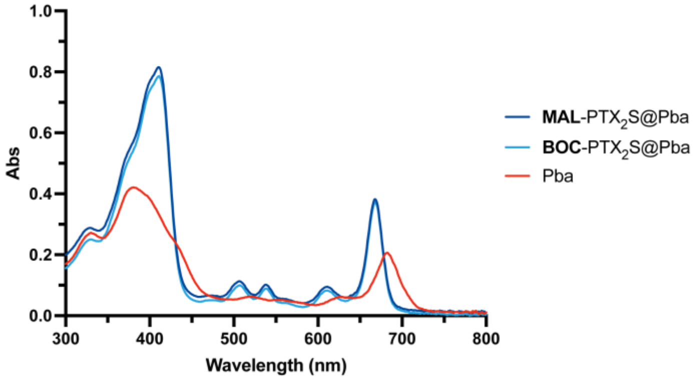

Figure S11. Absorption spectra of 10 µM Pba, **MAL-**PTX2S@Pba and **BOC**-PTX2S@Pba diluted in water.

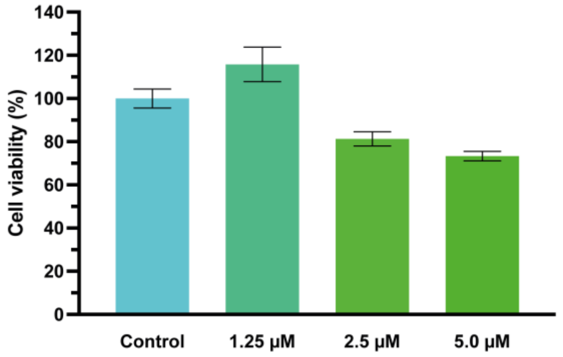

Figure S12. Cytotoxicity measured in 4T1 cells incubated with increasing concentrations of free PTX for 24h and release in drug-free medium for additional 48h before assessing cell viability with the MTS assay. Data are expressed as mean percentage ± SD of least three independent experiments, carried out in triplicate.

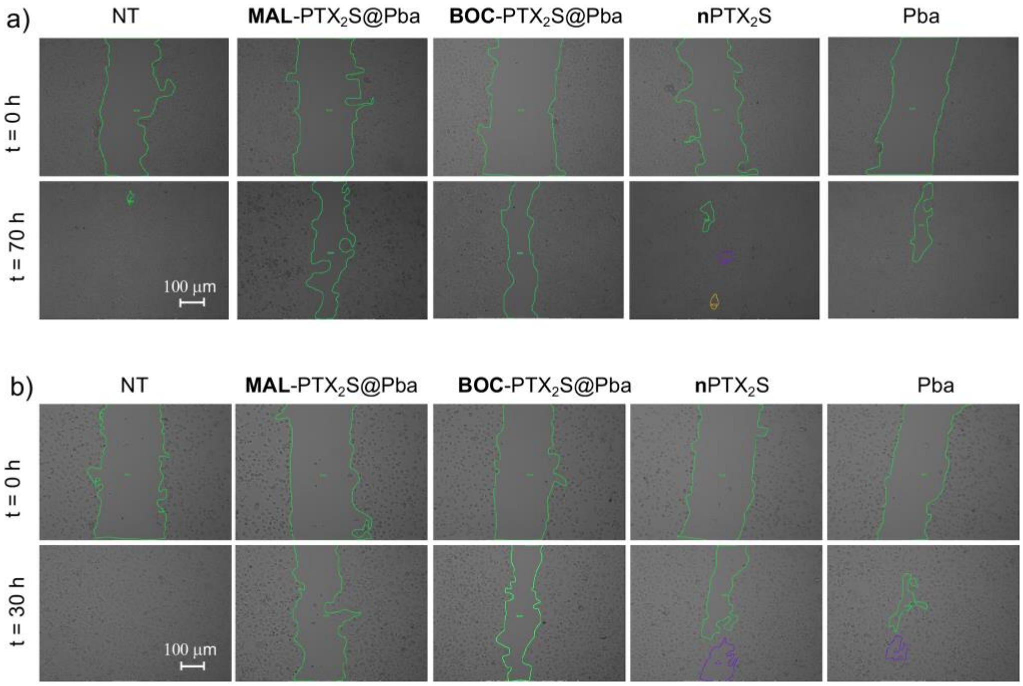

Figure S13. The motility of MCF-7 cells (a) and MDA-MB-231 cells (b) was measured by wound healing assay. Phase-contrast microscopy images (scale bar: 100 µM) at the beginning of the experiment (0h) and the end point (70h for MCF-7 and 30h for MDA-MB-231 cells).

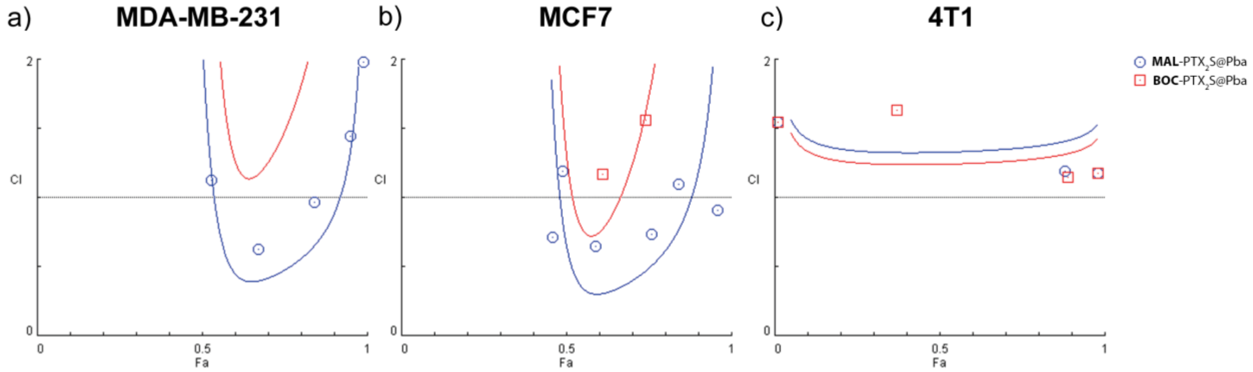

Figure S14. Combination index analysis. Fa-CI plots of Combination Index vs. Fa relative to MDA-MB-231, MCF-7 and 4T1 cells treated with the combination of PTX and Pba loaded in **MAL**-PTX_2S@Pba or **BOC**-PTX_2S@Pba. Data reported in Fig. 5 d-f of the main text were analyzed with the Compusyn software and Fa-CI plots derived.

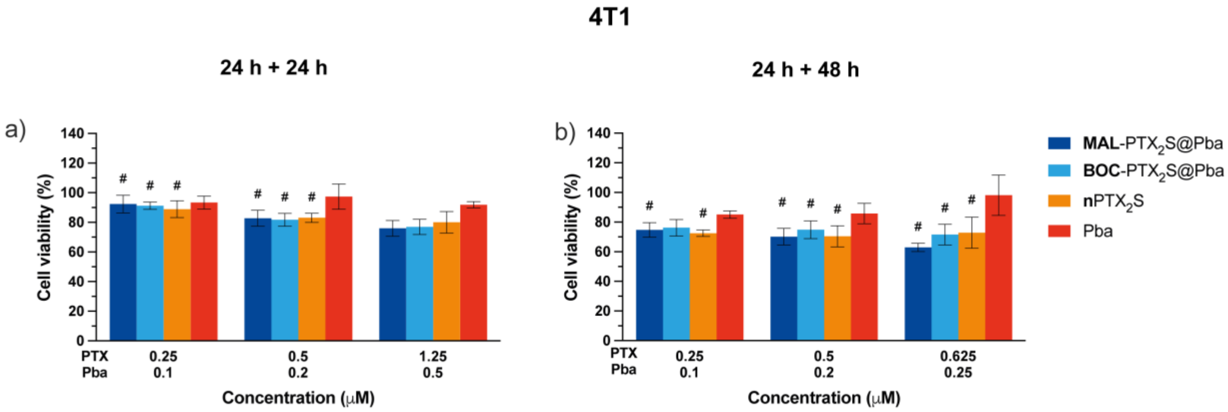

Figure S15. Dark cytotoxicity measured in 4T1 spheroids incubated with the different drugs/combinations for 24h and maintaned in drug-free medium for additional 24 (a) or 48 (b) h before assessing cell viability by measuring ATP levels with the 3D Glo assay. Data are expressed as mean percentage ± SD of at least two independent experiments, carried out in quadruplicate. Statistical significance was calculated applying the ANOVA Two-way with Bonferroni's correction: # significantly different from Pba.

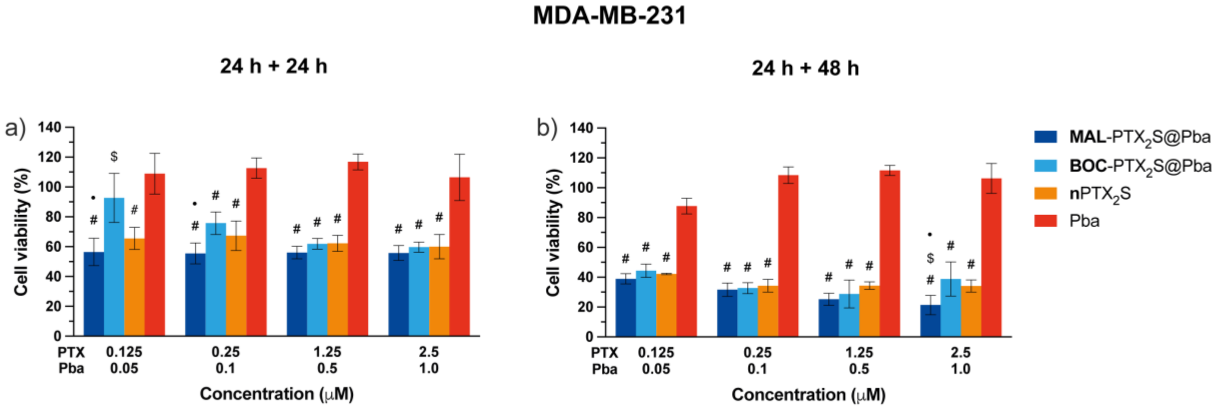

Figure S16. Dark cytotoxicity measured in MDA-MB-231 spheroids incubated with the different drugs/combinations for 24h and maintaned in drug-free medium for additional 24 (a) or 48 (b) h before assessing cell viability by measuring ATP levels with the 3D Glo assay. Data are expressed as mean percentage ± SD of at least two independent experiments, carried out in quadruplicate. Statistical significance was

calculated applying the ANOVA Two-way with Bonferroni's correction: • significantly different from **BOC**-PTX2S@Pba; # significantly different from Pba; $ significantly different from **n**PTX2S.

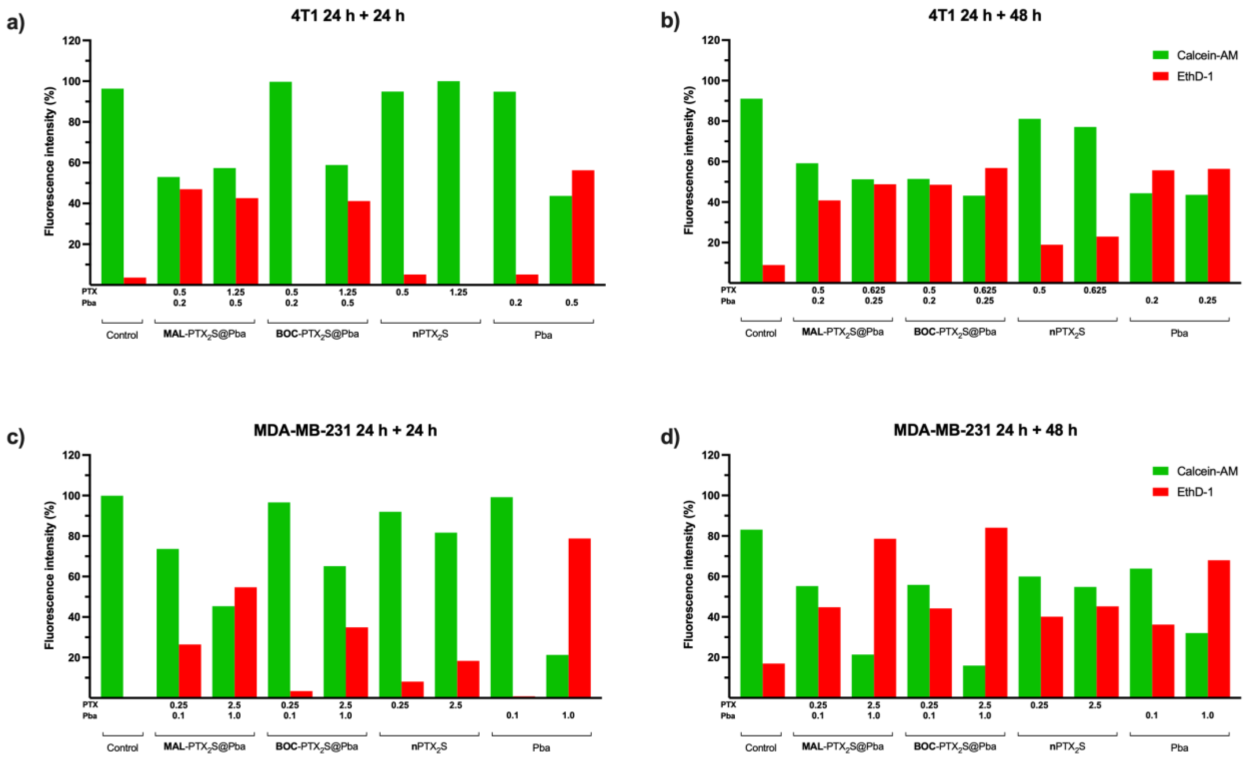

Figure S17. Quantification of fluorescence intensity in 4T1 and MDA-MB-231 spheroids stained with the LIVE/DEAD kit. The images reported in Figure 9 (4T1 spheroids) and 10 (MDA-MB-231 spheroids) were quantified using ImageJ software in order to quantify the fluorescence intensity from live cells (stained by calcein AM, green fluorescence) and dead cells (stained by Ethidium homodimer-1 (EthD-1), red fluorescence). a) quantification of images in Fig. 9 c; b) quantification of images in Fig. 9 d; c) quantification of images in Fig. 10 c; d) quantification of images in Fig. 10 d.

Table S4. IC_{50} values calculated by the Compusyn software from the photo-toxicity data (Figure 6 g,j) of MDA-MB-231 and 4T1 spheroids exposed to the different drug formulations/combinations and irradiated with 1 J/cm[2] of red light.

Drug formulation	IC_{50} (µM)		IC_{50} (µM)	
	**MDA-MB-231** 24+24 h	**4T1** 24+24 h	**MDA-MB-231** 24+48 h	**4T1** 24+48 h
**n**PTX_2S	-	-	0.12	-
Pba	0.27	0.16	0.33	0.16
**MAL**-PTX_2S@Pba	0.39	0.78	0.12	0.69
**BOC**-PTX_2S@Pba	1.17	0.83	0.11	0.58

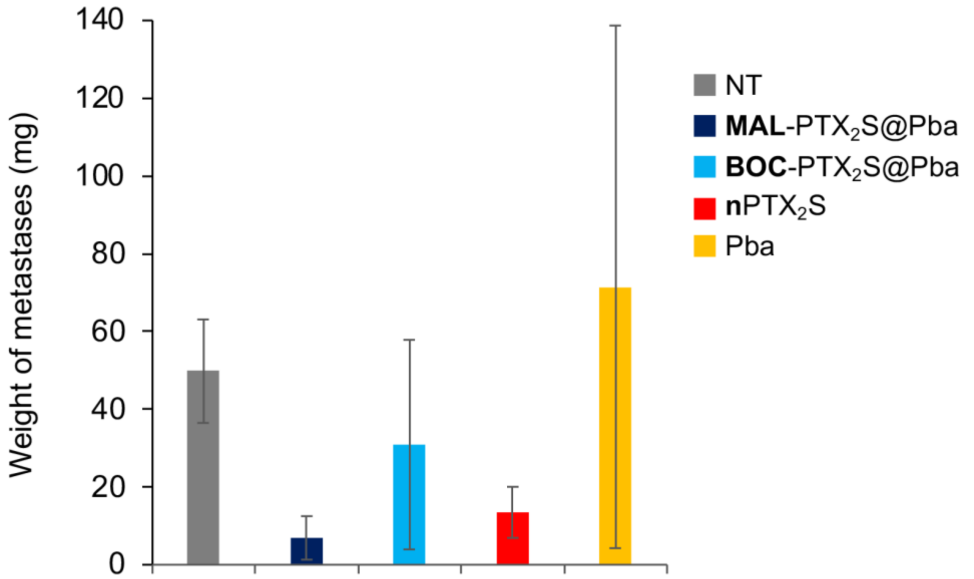

Figure S18. Antitumor efficacy on weight of lung metastases. At the sacrifice day, the lungs were harvested to examine weight of metastases by stereomicroscope. Metastasis mass (mg) was calculated assuming a density of 1 and a volume of π/6 x a2 x b, where a and b are the shorter and larger axes (cm), respectively. The results were expressed as the mean ± SE (standard error). A statistical difference was observed through the unpaired t-test between **MAL**-PTX_2S@Pba ($p=0.0038$), **BOC**-PTX_2S@Pba ($p=0.006$) and **n**PTX_2S ($p=0.003$) as respect to untreated group, as well as between **MAL**-PTX_2S@Pba and **BOC**-PTX_2S@Pba treatments ($p=0.007$).
